# Multiscale Defective Interfaces for Realizing Na‐CO_2_ Batteries With Ultralong Lifespan

**DOI:** 10.1002/adma.202409533

**Published:** 2024-10-09

**Authors:** Changfan Xu, Ping Hong, Yulian Dong, Yueliang Li, Yonglong Shen, Johannes Biskupek, Huaping Zhao, Ute Kaiser, Guosheng Shao, Yong Lei

**Affiliations:** ^1^ Fachgebiet Angewandte Nanophysik Institut für Physik & IMN MacroNano Technische Universität Ilmenau 98693 Ilmenau Germany; ^2^ Central Facility for Electron Microscopy Electron Microscopy Group of Materials Science Ulm University 89081 Ulm Germany; ^3^ School of Materials Science and Engineering Zhengzhou University Zhengzhou 450001 China

**Keywords:** CO_2_ batteries, CO_2_ cathode, defective interfaces, dendrite‐resistant, Na metal anode

## Abstract

Despite their favorable high energy density and potential for CO_2_ recycling, Na‐CO_2_ batteries have been held back by limitations in cycling capability, stemming from the sluggish CO_2_ reduction/evolution reaction (CO_2_RR/CO_2_ER) kinetics at CO_2_ cathode and unmanageable deposition/stripping of metallic Na at the anode upon cycling. Herein, a “two‐in‐one” electrode with multiscale defective FeCu interfaces (CP@FeCu) is presented, which is capable of improving the CO_2_RR/CO_2_ER kinetics of CO_2_‐breathing cathode, while modulating sodium deposition behavior. Experimental and theoretical investigations reveal multiscale defective FeCu interfaces are responsible for the enhancement of sodiophilicity and catalytic properties. The defect and valence oscillation effects originate in multiscale defective FeCu interfaces, effectively facilitating the adsorption of reactants and decomposition of Na_2_CO_3_ during CO_2_RR/CO_2_ER processes, along with exceptional cycling stability of 2400 cycles (4800 h) at 5 µA cm^−2^. Meanwhile, the CP@FeCu with sodium affinity creates a uniform electric field and robust adsorption for Na, making initial nucleation sites more conducive to Na deposition and achieving dendrite‐resistant and durable anodes. This work offers a scientific insight into the functionalization design of “two‐in‐one” electrodes, which is essential for a unified solution to the challenges in sodium anodes and CO_2_ cathodes.

## Graphical abstract

This work proposes a multiscale defective interface concept via designing a “two‐in‐one” electrode to simultaneously enhance sodiophilicity and CO_2_RR/CO_2_ER properties, facilitating the development of durable Na‐CO_2_ batteries. The CP@FeCu electrode functions as a CO_2_ cathode with exceptional cycling stability exceeding 2400 cycles. Additionally, it optimizes sodium deposition behavior as a sodium stripping/plating host, achieving a dendrite‐resistant and long‐lasting anode.

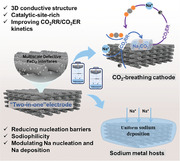

## 1 Introduction

Sodium‐carbon dioxide (Na‐CO_2_) batteries, based on a conversion reaction of 4Na + 3CO_2_ ↔ 2Na_2_CO_3_ + C, own a high energy density (1125 Wh kg^−1^) and are a friendly option to recycle greenhouse gas CO_2_ and store the electricity generated from renewable energy.^[^ ^1^ ^]^ Despite high expectations for Na‐CO_2_ batteries, their development is hindered by inherent limitations, generally resulting in limited lifespans.^[^ ^1^, ^2^, ^3^ ^]^ A fundamental reason for the limited lifespan can be attributed to the low catalytic activity of CO_2_ cathodes, which is insufficient to fully catalyze the decomposition of the inert Na_2_CO_3_ discharge products, resulting in continuous accumulation of Na_2_CO_3_ on the cathode. Excessive accumulation of Na_2_CO_3_ not only clogs the porous channels within the cathode for the diffusion of Na^+^ and CO_2_ but also passivates the active surfaces of the CO_2_ cathodes, hence resulting in poor cycle life.^[^ ^4^, ^5^, ^6^ ^]^ Moreover, the dissolution and deposition of highly chemically reactive metallic sodium on anode during multiple energy storage and release cycles can lead to drastic volume changes, dendritic sodium, or inactivated “dead” sodium, which can easily lead to poor durability or even rapid failure of the battery.^[^ ^7^, ^8^, ^9^ ^]^ Excess sodium foil is commonly included in Na‐CO_2_ batteries to compensate for the loss of Na during the battery operation, but this introduction of additional Na is not conducive to the intended application of these batteries and does not contribute at all to solve the dendrite formation problem.^[^ ^10^, ^11^ ^]^ How to address these challenges on cathode and anode simultaneously provides a timely and crucial topic.

To date, a variety of strategies have been proposed to address the problems encountered with the two electrodes mentioned above. Regarding CO_2_ cathodes, research endeavors have primarily concentrated on spraying the designed catalyst onto a conductive carbonaceous gas diffusion layer to facilitate rapid redox reactions through modulation of both the apparent physical and intrinsic electronic structures of the catalyst.^[^ ^12^, ^13^ ^]^ For the anode, strategies include customizing the electrolyte,^[^ ^11^ ^]^ building a stable NaF‐rich SEI layer,^[^ ^14^ ^]^ modifying a sodium‐containing composite anode,^[^ ^3^ ^]^ or using a solid‐state electrolyte^[^ ^7^, ^8^ ^]^ to construct a stable sodium flux and inhibit the generation of sodium dendrites. Nevertheless, most of the current studies are limited to unilateral electrodes, which cannot fundamentally solve the aforementioned problems faced by Na‐CO_2_ batteries. Crucially, the anode and cathode should be organized systematically rather than merely considering individual electrodes. Therefore, it remains desirable to strategically design configurations for both the CO_2_ cathode and Na anode to synchronize and effectively address the aforementioned challenges in advanced Na‐CO_2_ systems. However, such research for the Na‐CO_2_ battery field has yet to yield breakthroughs.

To obtain bidirectional electrodes with highly efficient active sites for both CO_2_RR/CO_2_ER and sodium deposition, a “two‐in‐one” design concept is to establish sodiophilic and CO_2_ redox sites onto the surface of 3D porous host structures. In general, the phenomenon of “atomic size misfit” can give rise to lattice distortions, dislocations, and interfacial defects,^[^ ^15^, ^16^, ^17^, ^18^, ^19^, ^20^ ^]^ which invariably impact the distribution of lattice stress, surface charges on the electrode, and subsequent interfacial reactions, such as sodium deposition/stripping and CO_2_ conversion. Understanding the relationship between the defect structures of the host material and the battery performance at the atomic defect level is of importance for the optimization of CO_2_ redox and sodium deposition behavior.

Taking these considerations into account, we put forward here a multiscale defective interface concept via atomic misfit‐driven reconfiguration to design Cu‐doped iron oxide nanoparticles grown on carbon paper electrode (CP@FeCu), serving as a CO_2_‐breathing cathode and an efficient Na deposition host for Na‐CO_2_ batteries. In particular, CP@FeCu as a CO_2_‐breathing cathode achieves an ultralong cycle of ≈2400 cycles (4800 h) at 5 µA cm^−2^, which is much better than that of all reported cathodes in Na‐CO_2_ batteries. Moreover, CP@FeCu as an anode host also offers an ultralong sodium stripping/plating lifetime. Microstructural characterization shows that CP@FeCu is featured by multiscale defective interfaces, including atomic‐level Cu defects, twin boundaries, nanoscale mosaicking, vacancies, and lattice distortions. The multiscale defects induce the change of d‐band centers and the oxidation state of Cu, regulating the adsorption/decomposition of intermediates during CO_2_RR/CO_2_ER processes, and enhancing catalytic performance. Meanwhile, the defective interfaces provide an electron‐rich environment and enhanced adsorption for sodium, which lower the nucleation energy barrier and lead the sodium ions to be uniformly deposited in the host framework, facilitating a dendrite‐free morphology. The understanding based on the proposed CP@FeCu bidirectional electrode provides a solution for realizing long‐lived Na‐CO_2_ batteries through multiscale defective interfaces, which could potentially advance the possible future application of Na‐CO_2_ batteries.

## 2 Results and Discussion

### 2.1 DFT Pre‐Analysis for “Two‐In‐One” Electrodes

**Figure 1:**
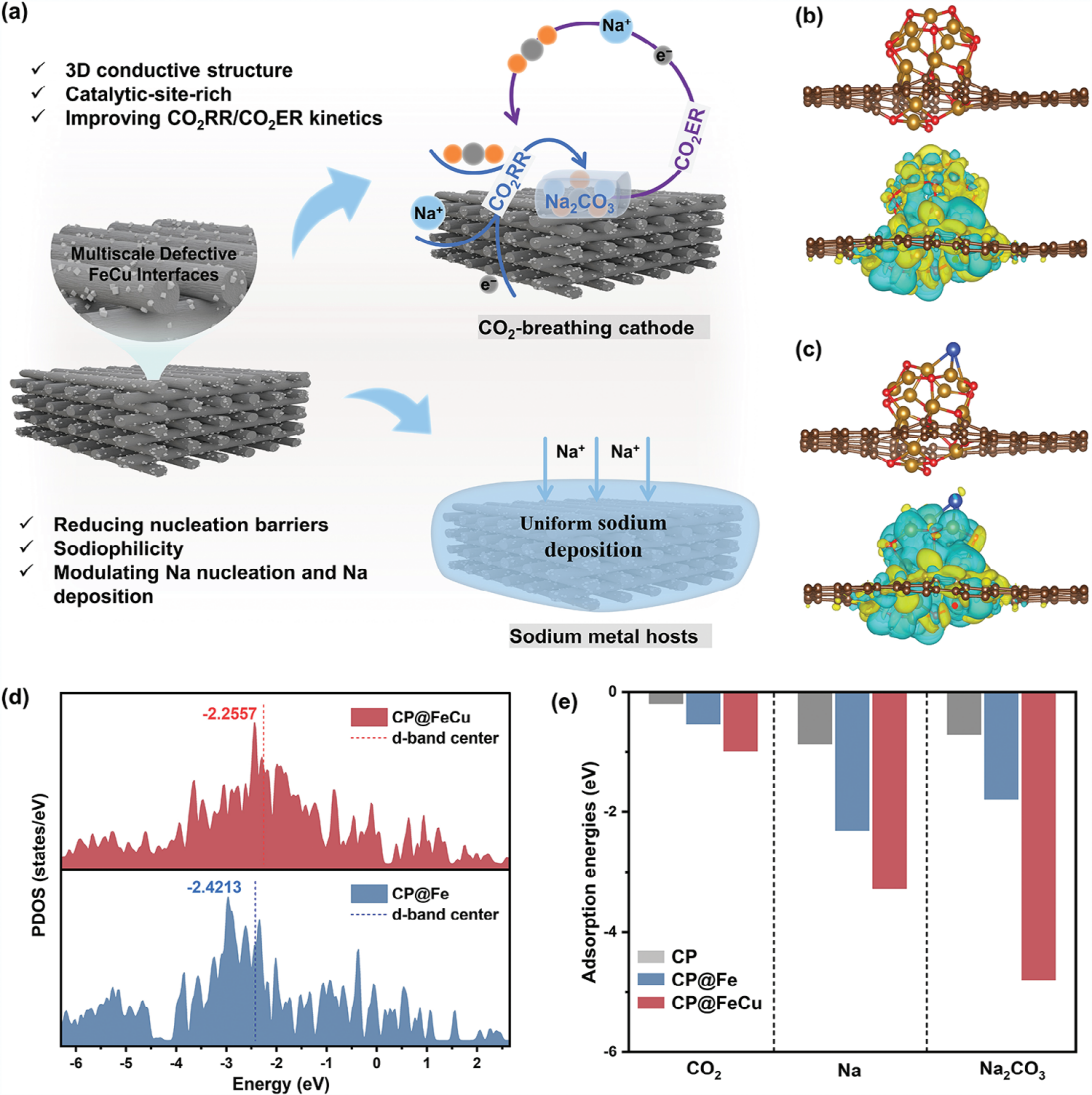
a) Schematic illustration of a “two‐in‐one” electrode designed to promote CO_2_ conversion and regulate the nucleation and growth of sodium. b,c) Optimized structures and corresponding charge density difference of pristine CP@Fe and CP@FeCu interface (Brown: C; Dark yellow: Fe; Blue: Cu; Red: O). Cyan and yellow colors represent losing and gaining electrons, respectively. The isosurface level was set to be 0.001 e Å^−3^. d) Partial density of states (PDOS) of CP@Fe and CP@FeCu interface. e) Adsorption energies of CO_2_, Na, and Na_2_CO_3_ on CP, CP@Fe, and CP@FeCu interfaces.

We aim to design a bidirectional electrode that harnesses a 3D structure with consistent ion/electron transport kinetics to achieve an exceptional duration for Na‐CO_2_ batteries. Certainly, a bidirectional electrode should fulfill the following roles (**Figure** **1**a). On the anode side, the electrode can act as a sodiophilic host with low nucleation barriers to build a homogeneous sodium flux to promote the Na^+^ ions migration throughout the entire host for uniform deposition. On the cathode side, the electrode can directly act as a self‐supporting cathode with catalytic activity that can promote fast CO_2_RR/CO_2_ER kinetics. Therein, density‐functional theory (DFT) calculations were first performed to establish the theoretical models of electrodes comprising carbon paper‐supported Cu‐doped iron oxide nanoparticles (CP@FeCu), as well as their corresponding single‐component electrodes (CP@Fe and CP). A simplified model of a Fe_3_O_4_ cluster doped with a single copper atom was built reasonably. This model captures the interactions between Fe and Cu and allows us to study the fundamental role of the embedded Cu atom in modulating the electronic properties of the Fe_3_O_4_ matrix, influencing the reaction pathways, energy barriers, and overall catalytic activity. Figures 1b,c demonstrate the atomic structures and corresponding charge density difference of CP@Fe and CP@FeCu interfaces. The redistribution of charge is promoted by copper doping, and the interfacial charge is transferred from the Fe_3_O_4_ clusters to the carbon substrate, indicating a strong interaction at the CP@FeCu interface. Figure 1d shows the partial density of states (PDOS) of CP@Fe and CP@FeCu interfaces. The d‐band center, widely regarded as a reliable indicator of the adsorbate‐metal interaction,^[^ ^21^ ^]^ exhibits a higher value on the CP@FeCu interface compared to that on the CP@Fe interface (−2.2557 vs −2.4213 eV). The upward shift of the d‐band center may enhance the interaction between Na^+^ ions, CO_2_ molecules, and the CP@FeCu interface, which is conducive to the enhancement of Na adsorption energy during sodium deposition and the adsorption capacity of intermediates during the CO_2_RR process.

To provide a better understanding of sodium deposition and CO_2_RR kinetics, we conducted an additional investigation on the adsorption configurations of Na and CO_2_ as well as the associated charge density differences on the three substrates (Figures S1–S4, Supporting Information). The findings regarding adsorption energy are summarized in Figure 1e. Na adsorption energy is found to be significantly stronger for −2.30 and −3.27 eV on CP@Fe and CP@FeCu interfaces, respectively, compared to that of pure CP (−0.83 eV). This result implies the presence of effective sites with a strong affinity for sodium in CP@Fe and CP@FeCu, facilitating the stabilization of Na embryo formation by reducing the energetic barrier to nucleation. The nucleation of Na at the CP@FeCu interface is, in particular, energetically favorable. In addition, the CP@FeCu system exhibits a pronounced charge reconstruction effect, resulting in enhanced adsorption of C and O atoms onto Fe and Cu, it is noteworthy that the adsorption energy of CO_2_ on CP@FeCu surpasses that of both CP@Fe and CP. This suggests that activating CO_2_ reduction becomes more feasible with CP@FeCu.

The CP@FeCu demonstrates promising capabilities in enhancing CO_2_RR kinetics due to its efficient electron transfer and robust adsorption sites for CO_2_ and Na, thereby contributing to the nucleation and uniform distribution of Na_2_CO_3_ during CO_2_RR. However, it is crucial to achieve efficient decomposition of Na_2_CO_3_ for CO_2_ER. Calculations indicate that the bond length of Na─O in Na_2_CO_3_ undergoes modifications upon interaction with CP@Fe and CP@FeCu (Figure S5, Supporting Information). The electron transfer between Fe and Cu within the CP@FeCu structure likely influences the charge distribution of Na_2_CO_3_ molecules, leading to a more noticeable enlargement of Na─O bonds compared to CP@Fe. Meanwhile, the differential charge density analysis reveals a trend of increasing electron loss in the Na─O bonds from CP to CP@Fe and then to CP@FeCu, indicating a decreasing strength of the Na─O bonds and favoring the decomposition of Na_2_CO_3_ (Figure S6, Supporting Information). These results suggest that Na_2_CO_3_ can be activated more easily in CP@FeCu to promote CO_2_ER.

### 2.2 Synthesis and Characterization of CP@FeCu

Atomic misfit‐driven CP@FeCu were fabricated from Prussian blue analogs through in situ preparation and thermal treatment (see details in Supporting Information). For comparison, a CP@Fe electrode was prepared without Cu salts. Scanning electron microscopy (SEM), and transmission electron microscopy (TEM) images show that the precursor, FeCu Prussian blue analogues, exhibits a nanocubic morphology with an average size of 200–300 nm (Figure S7, Supporting Information). The powder X‐ray diffraction (XRD) pattern of Cu‐FeO*_x_* for CP@CuFe electrode displays copper lattice features, with peaks located at 43.3°, 50.5° and 74.2° corresponding to the (111), (200), and (220) planes of Cu (PDF: 70–3039), in comparison to that of CP@Fe electrode (Figure S8, Supporting Information). Moreover, peaks located at 35.4°, 57.0°, and 62.6° could be ascribed to the (311), (511), and (440) planes of Fe_3_O_4_ (PDF: 75–0033), which are slightly shifted to the low‐angle region, indicating a slight expansion of the lattice of the Fe_3_O_4_ nanoparticles. A significant difference in the morphology of the CP@FeCu and CP@Fe is observed by SEM and TEM (Figures S9–S11, Supporting Information), demonstrating that Cu doping leads to the formation of small‐sized morphology. High‐resolution TEM (HRTEM) of CP@Fe (Figure S10, Supporting Information) reveals the presence of carbon‐coated particles, with the carbon originating from the thermal decomposition of CN^−^ salt during heat treatment. This result is corroborated by the analysis of energy dispersive spectroscopy (EDS) elemental mapping (Figure S12, Supporting Information). Moreover, the lattice arrangement is well‐defined and regular throughout the entire particle, with the lattice spacing of 0.294 nm corresponding to the (220) crystal plane of Fe_3_O_4_ (Figure S10, Supporting Information). However, in CP@FeCu, such lattice fringes are significantly broken and distorted (Figure S11, Supporting Information), and various defects can be observed, including lattice distortion, atomic vacancies, grain boundaries (GBs), and nanoscale mosaic defect structures within the grains grown on the main matrix. These defects and interfaces in the CP@FeCu composite could contribute together to enhance electron transfer and upward shift of the d‐band center, exhibiting unique electronic properties not found in pristine CP and CP@Fe.

**Figure 2:**
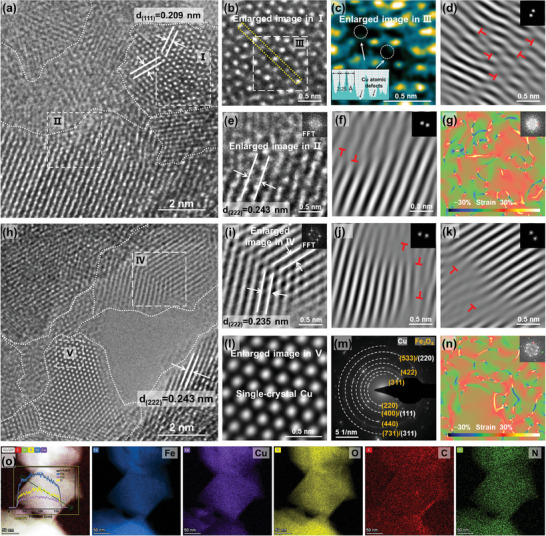
a,h) Representative Cc/Cs‐corrected HRTEM images of CP@FeCu, with abundant GBs marked with white dashed lines. b,e) Enlarged figures in a. The inset in e shows the corresponding Fourier transform (FFT) pattern. c) Enlarged figure in b, and intensity profiles labeled in b with yellow dashed boxes. d,f) Inverse fast Fourier transform (IFFT) pattern of b,e. g) Strain distributions of e_xy_ in a. i,l) Enlarged figures in h, indicating the presence of twinned and single‐crystal nanoparticles. The inset in i shows the corresponding FFT pattern. j,k) IFFT patterns of i. m) SAED pattern. n) Strain distributions of e_xy_ in h. o) HAADF‐STEM image and EDS maps, and corresponding line‐scan profile.

The representative Cc/Cs‐corrected HRTEM images provide a direct understanding of how defect structures form in the CP@FeCu matrix at the atomic level. Specifically, the CP@FeCu surface exhibits a densely packed structure of nanoparticles with nanometer‐sized grains and an abundance of GBs (**Figures** **2**a,h). The region I depicted in Figure 2a illustrates a twinned structure of Cu nanoparticles, accompanied by lattice fringes exhibiting d‐spacings of 0.209 nm corresponding to the (111) plane of the Cu phase. Enlarged images (Figures 2b,c) and the variation in the line intensity distribution of Cu–Cu distance corresponding to the selected yellow dashed area confirm the existence of Cu atomic defects. Region II enlarged image (Figure 2e) reveals a lattice spacing of 0.243 nm, indicative of the (222) plane of Fe_3_O_4_. Further analysis indicates that these nanoparticles also exhibit characteristics of other twinned nanoparticles, with a lattice distance of 0.235 nm that could be indexed to the (222) crystal plane of CuO or Fe_3_O_4_ phases (Figure 2i), as well as features of single‐crystal Cu, where the Cu‐Cu atomic distance is measured at 2.29 Å (Figure 2l). Additionally, the highly distorted multiscale defect is also elucidated by the inverse fast Fourier‐transform (IFFT) analysis, as evidenced by the abundant disordered lattice structures with crystallographic defects and edge dislocations (Figures 2d,f,j,k). Lattice distortion/strain is also evidenced by geometric phase analysis of the entire region shown in Figures 2a,h. As illustrated in Figures 2g,n, the resulting shear strain distribution (e_xy_) reveals that the strain predominantly propagates along GBs and defect sites, providing further evidence of lattice defects in CP@FeCu. The diffraction patterns in Figure 2m from the selected area electron diffraction (SAED) image indicate the presence of Fe_3_O_4_ and Cu species, suggesting that these nanoparticles exhibit polycrystalline phases. Furthermore, the outcomes derived from element mapping and line‐scan profile (Figure 2o) demonstrate a uniform distribution of Fe and Cu within the CP@FeCu matrix, and the active materials are mixed with nitrogen and carbon.

### 2.3 Investigating the Sodiophilicity of CP@FeCu

To experimentally validate the sodiophilic properties of the CP@FeCu obtained, the nucleation overpotentials of several substrates were assessed. In this case, 1 m sodium hexafluorophosphate (NaPF_6_) in diglyme was preferred as a reliable electrolyte for Na plating/stripping tests in an asymmetric cell configuration due to its lower viscosity, higher ionic conductivity, superior solvation ability, and better interfacial compatibility compared to 1 m sodium perchlorate (NaClO_4_) in tetraethylene glycol dimethyl ether (TEGDME).^[^ ^22^ ^]^ As depicted in Figure S13 (Supporting Information), all voltage profiles exhibit an initial drop during Na plating, succeeded by a consistent voltage plateau at 1 mA cm^−2^. Within this context, the overpotentials observed during Na deposition are distinguished as nucleation overpotentials (η_n_), denoting the difference in potential between the sustained plateau and the minimal voltage decrease, and plating overpotentials controlled by mass transfer (η_p_), linked to Na growth following the initial nucleation stage.^[^ ^23^ ^]^ Compared to Cu electrodes, CP‐based electrodes demonstrated decreased nucleation overpotentials, suggesting a diminished barrier for heterogeneous nucleation on these substrates due to the high affinity between sodium metal and them. Significantly, the CP@FeCu demonstrates the most minimal nucleation overpotential (≈13 mV) in comparison to CP and CP@Fe, highlighting the effectiveness of such a designed current collector in reducing the energetic barrier for sodium nucleation. The increased affinity toward sodium is primarily attributed to the numerous sites for nucleation present on the surface of CP@FeCu. In this regard, the incorporation of FeCu defective interfaces into CP proves advantageous in facilitating electron transfer to Na^+^ and ensuring homogeneous Na nucleation.

**Figure 3:**
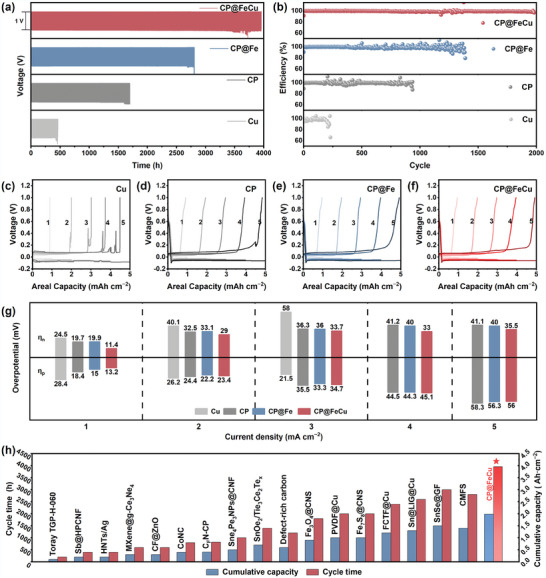
Electrochemical characterization of Na metal batteries. a) Long‐term cycling performances at 1 mAh cm^−2^ and 1 mA cm^−2^. b) corresponding Coulombic efficiency plots. c–f) Voltage profiles of Na plating/stripping at various areal current densities. g) Histograms of Na nucleation and plating overpotentials. h) Comparison of cyclic reversibility and cumulative capacity between this work and other previous reports.

The investigation also encompassed the plating and stripping behaviors of Na on different hosts. The activation procedures were performed before assessing the longevity of Na deposition to eliminate potential surface impurities and establish the initial SEI layer (Figures S14 and S15, Supporting Information). The voltage‐time profiles of different hosts are depicted in **Figure** **3**a, while the detailed features of charge–discharge curves at 1 mA cm^−2^ for various samples during the final cycles can be found in Figure S16 (Supporting Information). The Na plating/stripping behavior of CP@FeCu remains stable for ≈3960 h, far surpassing the endurance of bare Cu foil which could only last for 460 h due to short circuits. The stability of the CP@Fe electrode is somewhat improved, but it is still considerably less stable than the CP@FeCu electrode. This highlights the significance of incorporating a multiscale defective FeCu interface and creating sodiophilicity.

Furthermore, Coulombic efficiency (CE) serves as a crucial parameter for assessing the stability of Na‐metal anodes. Note that the CE discussed here refers to the ratio of capacity achieved during charging to discharging, including contributions from both plating/stripping and sodiation/desodiation processes. As seen in Figure 3b, CP@FeCu demonstrates remarkable cycling stability, completing 1980 cycles with a high average CE of ≈99.1%, a notable improvement compared to the other counterparts. Inspiringly, we have achieved significant improvements in stabilizing the cycle life of Na metal batteries by means of multiscale defective FeCu interfaces modified hosts in ester‐based electrolytes, compared to other sodiophilic coating techniques,^[^ ^24^, ^25^, ^26^, ^27^, ^28^, ^29^, ^30^, ^31^, ^32^, ^33^ ^]^ carbon skeletons,^[^ ^34^, ^35^, ^36^, ^37^, ^38^, ^39^ ^]^ or metallic scaffolds^[^ ^40^, ^41^ ^]^ as summarized in Figure 3h and Table S1 (Supporting Information). The evident disparities among these hosts highlight the advantage of the sodiophilic CP@FeCu in facilitating stable plating/stripping processes. Aurbach CE tests^[^ ^42^ ^]^ (Figure S17, Supporting Information) were also conducted to assess sodium cycling efficiency on CP, CP@Fe, and CP@FeCu hosts, which further proves the benefit of the CP@FeCu design by showing a substantially improved CE of 99.3% compared with CP@Fe (98%), and CP (96.9%). It is noted that CP exhibited a low activation voltage in the initial pre‐activation stage, which could be associated with the nature of the sodium ions inserted into the graphitized carbon fibers. Before plating, a large amount of Na⁺ has already been inserted into the graphitized carbon fibers, which leads to a relatively small nucleation barrier for subsequent Na plating, thus resulting in a low activation voltage during the initial pre‐activation phase. This insertion process consumes a large amount of Na^+^, giving rise to the low ratio of Na participated for plating/stripping.^[^ ^43^ ^]^ After initial activation, CP@FeCu exhibits excellent performance in terms of reduced nucleation overpotential and efficient Na deposition/stripping with higher coulombic efficiency due to its structural optimization to suppress Na^+^ loss caused by the insertion reaction before plating. Moreover, CP@FeCu also exhibits remarkable rate capability across a range of current densities spanning from 1 to 10 mA cm^−2^ (Figure S18, Supporting Information). The CP@FeCu electrodes exhibit steady and smooth voltage curves across various current densities (Figures 3c–f), featuring significantly lower values for both η_n_ and η_p_ compared to similar counterparts (Figure 3g). Figure S19 (Supporting Information) illustrates the cyclic voltammetry (CV) curves of asymmetric Na cells employing CP, CP@Fe, and CP@FeCu hosts, within the voltage range of −0.2–1 V and a sweep rate of 0.1 mV s^−1^. The CP@FeCu electrode exhibits a higher peak current compared to the CP and CP@Fe electrodes, suggesting that the CP@FeCu defective interfaces enhance the charge transfer kinetics during sodium deposition. Figure S20 (Supporting Information) presents the electrochemical impedance spectroscopy (EIS) of asymmetric Na cells with CP, CP@Fe, and CP@FeCu electrodes. The results show that the interfacial resistance of the CP@FeCu electrode is significantly lower than that of the CP and CP@Fe electrodes, providing direct evidence that the introduction of copper promotes the rapid migration of sodium ions and facilitates a low‐impedance Na plating/stripping process. As shown in Figure S21 (Supporting Information), electron spin resonance (ESR) revealed the presence of oxygen vacancies. The oxygen vacancy concentration in CP@FeCu is much higher than that of CP@Fe, suggesting that the introduction of copper leads to an increase in the number of defective sites, thereby augmenting the propensity for Na migration. Furthermore, to emulate the conditions of an initial‐anode‐free cell environment, we conducted additional investigations on the cycling stabilities of Na metal anodes with a higher amount of excess Na predeposited onto various hosts (four times the normal amount). Once all the pre‐plated excess Na metal has been depleted, the stripping curves would exhibit a typical plating/stripping behavior (Figure S22, Supporting Information). As shown in Figure S23 (Supporting Information), CP@FeCu exhibits high cycling stability, where 400% of the excess Na metal was completely consumed after 792 plating/stripping cycles, indicating that a high CE of 99.5% was achieved, indicating that the utilization of CP@FeCu effectively inhibits the irreversible consumption of Na metal and enhances the stability of the electrode–electrolyte interface.

**Figure 4:**
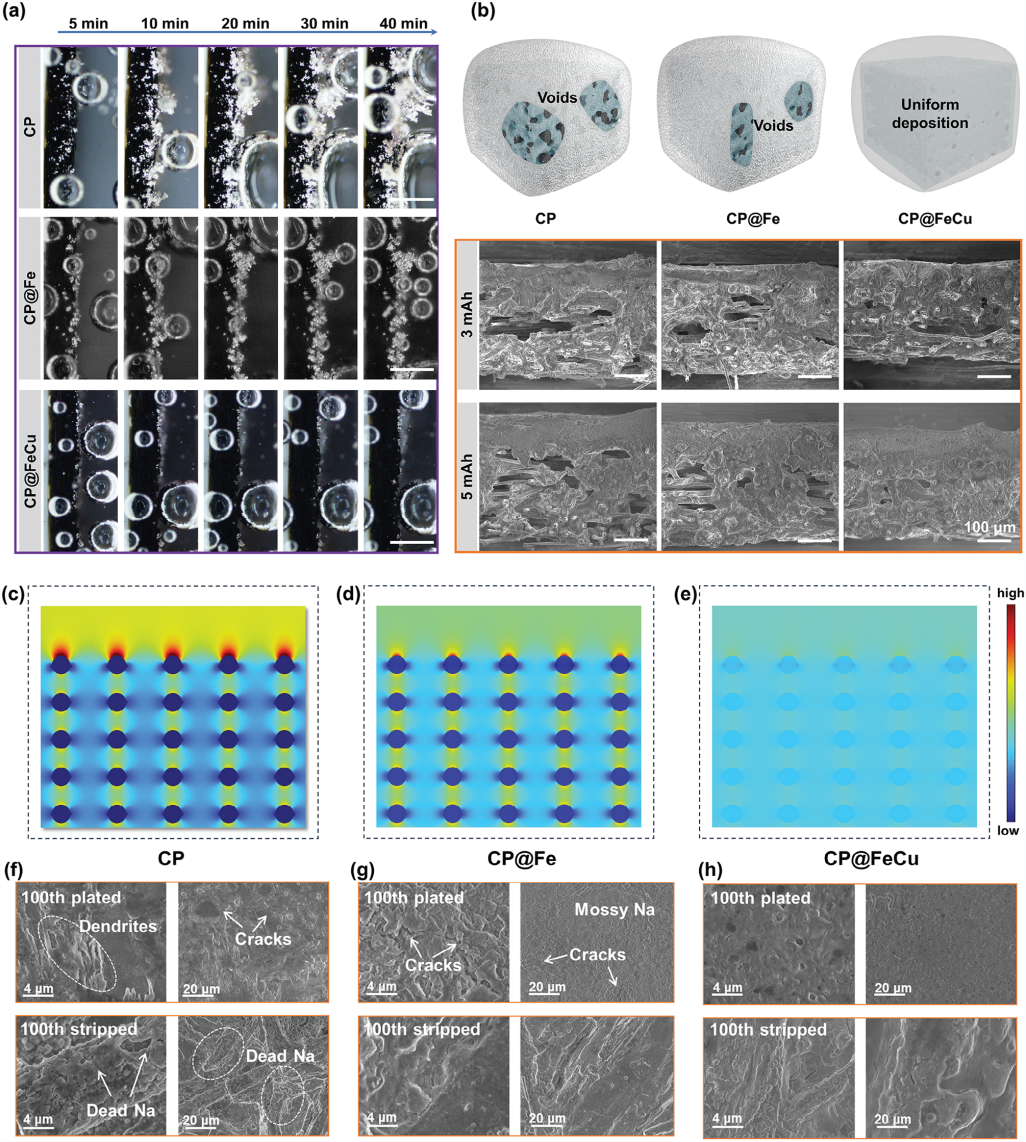
Physicochemical and theoretical investigation of Na metal deposition for three different hosts. a) In situ optical microscopy images of Na deposition, scale bars: 500 µm. b) SEM cross‐sectional images of Na metal deposited at different capacities, scale bars: 100 µm. COMSOL Multiphysics simulations c–e) E‐field distributions. f–h) SEM images after the 100th plating and 100th stripping.

To delve into the underlying factors governing the electrochemical performance of CP@FeCu, a more direct approach was employed to scrutinize the surface morphology of sodium metal. As depicted in **Figure** **4**a, the surface morphology evolution of various hosts during Na plating was initially visualized using in situ optical microscopy. After 5 min of plating, glistening silver‐white sodium nuclei seeds emerged on the surface of the CP electrode, exhibiting an uneven growth of sodium nuclei and remarkably rapid growth of dendrites, which were distinctly discernible (Movie S1, Supporting Information). Sodium nuclei grew more uniformly on the CP@Fe electrode compared to the CP electrode, but dendrites also appeared after 5 min (Movie S2, Supporting Information). As the amount of plated Na increases, the dendrites grow radially and gradually cover the whole surface of CP and CP@Fe electrodes. In marked contrast, hardly any sodium dendrites were observed during Na plating on the CP@FeCu surface, suggesting that CP@FeCu effectively promotes homogeneous deposition of Na without agglomeration, resulting in a dendrite‐free Na anode (Movie S3, Supporting Information).

The plated hosts were also subjected to ex situ SEM measurements to observe their morphologies. In the case of bare CP electrodes (Figure S24, Supporting Information), due to uneven flux distribution and poorly sodophilic interfaces, unevenly distributed large‐sized Na nuclei formed on their surfaces when discharged up to 0.5 mAh cm^−2^. As deposition proceeds, moss‐like dendrites appear, presenting a highly loose and porous surface when deposited up to 3–5 mAh cm^−2^. The issues of dendrites and surface porosity in CP@Fe are somewhat alleviated, yet they remain unresolved to perfection, which results in a homogeneous yet rough surface (Figure S25, Supporting Information). However, the phenomenon is different for CP@FeCu (Figure S26, Supporting Information), where Na is tightly packed and covered in carbon fibers under 0.5 mAh cm^−2^ deposition conditions. The Na metal gradually fills the spaces between the fibers as its capacity increases, and then it expands laterally, eventually forming a flat and smooth surface. Cross‐sections of the three electrodes plated at varying sodium deposition capacities confirm the uniform distribution of Na in the CP@FeCu hosts (Figure 4b). The presence of numerous voids on the CP and CP@Fe hosts is evident, suggesting that metallic Na readily deposits on the surfaces of CP and CP@Fe. In contrast, CP@FeCu exhibits dense Na deposition with a significantly smaller surface thickness compared to the other two hosts. This phenomenon suggests that Na metal on CP and CP@Fe often fails to deposit uniformly due to the lack of effective guidance, resulting in the presence of a large number of voids, and CP@FeCu provides a unique defective interface for the uniform deposition of Na, ensuring the dense and flat Na deposition morphology and effectively accommodating volume expansion.

To establish a connection between electrode structure and charge distribution, our approach commenced with a simulation of electric field distribution across the three electrodes using COMSOL Multiphysics software (Figures 4c–e). Notably, the upper layer of bare CP demonstrated pronounced electric field polarization in contrast to the modified CP electrode, resulting in a notable shielding effect that hinders sodium deposition. In sharp contrast, the electric field distribution within the CP@FeCu host exhibited remarkable uniformity compared to CP and CP@Fe hosts, fostering the creation of an equipotential surface that effectively curtails charge accumulation. Moreover, we delved into the evolution of Na morphology during the deposition process. As depicted in Figure S27 (Supporting Information), the enhanced localized electric field strength on the pristine CP substrate creates a region of high charge that drives a concentrated flux of sodium ions in the top layer, which instigates sodium nucleation and growth in the upper layer of CP substrate, resulting in a large number of voids in the substrate bulk. For comparison, the modified CP@Fe substrate exhibits relatively uniform deposition, unfortunately, sodium still subsequently aggregates and grows in the upper layers of the CP@Fe substrate. With the advantage of having the ability to promote electron transfer and homogenize the local current density, CP@FeCu effectively avoids the accumulation of high current density, resulting in the gradual and uniform deposition of sodium metal across the entire substrate and, consequently, the attainment of a stable sodium metal anode. In addition, our experiments have provided additional validation for these simulations. As demonstrated in Figures 4f–h, the characterization of the post‐cycling electrodes further validates the outstanding capability of CP@FeCu in suppressing dendrite formation and stabilizing the metal electrode. For bare CP, after the 100th deposition, a substantial amount of mossy fiber‐like dendrites and cracks appeared. Upon the 100th stripping, the electrode retained numerous inactive and non‐removable “dead Na”, indicating poor cyclic stability of Na metal on bare CP. Although there is some improvement with CP@Fe, cracks and Na whiskers are observed on the surface of the deposits. In distinct contrast, the plating/ stripping of Na metal in CP@FeCu still resulted in a flat and uniform interface even after 100 cycles, indicating that the CP@FeCu substrate possesses an exceptional affinity for sodium, thereby bestowing superior stability upon the Na anode. Consequently, the morphological characterization and computational results indicate that CP@FeCu can well achieve uniform nucleation and deposition of Na by providing uniform affinity for Na sites and regulating Na^+^ flux within the host structure.

### 2.4 Investigating the Catalytic Properties of CP@FeCu for CO_2_RR/CO_2_ER

**Figure 5:**
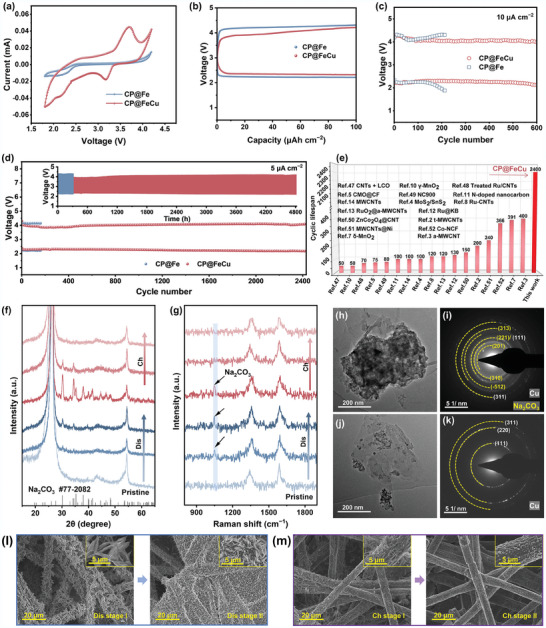
Electrochemical performances and discharge/charge mechanism of CP@FeCu as cathode for Na‐CO_2_ batteries in aprotic electrolyte. a) CV curves. b) The initial discharge‐recharge curves at 5 µA cm^−2^. Long‐term cycling performances at c) 10 µA cm^−2^ and d) 5 µA cm^−2^. e) the comparison of the battery performance with other works. f) Ex situ XRD profiles and g) Raman spectra, h,j) TEM and i,k) corresponding SAED, as well as l,m) SEM images after discharge and recharge.

**Figure 6:**
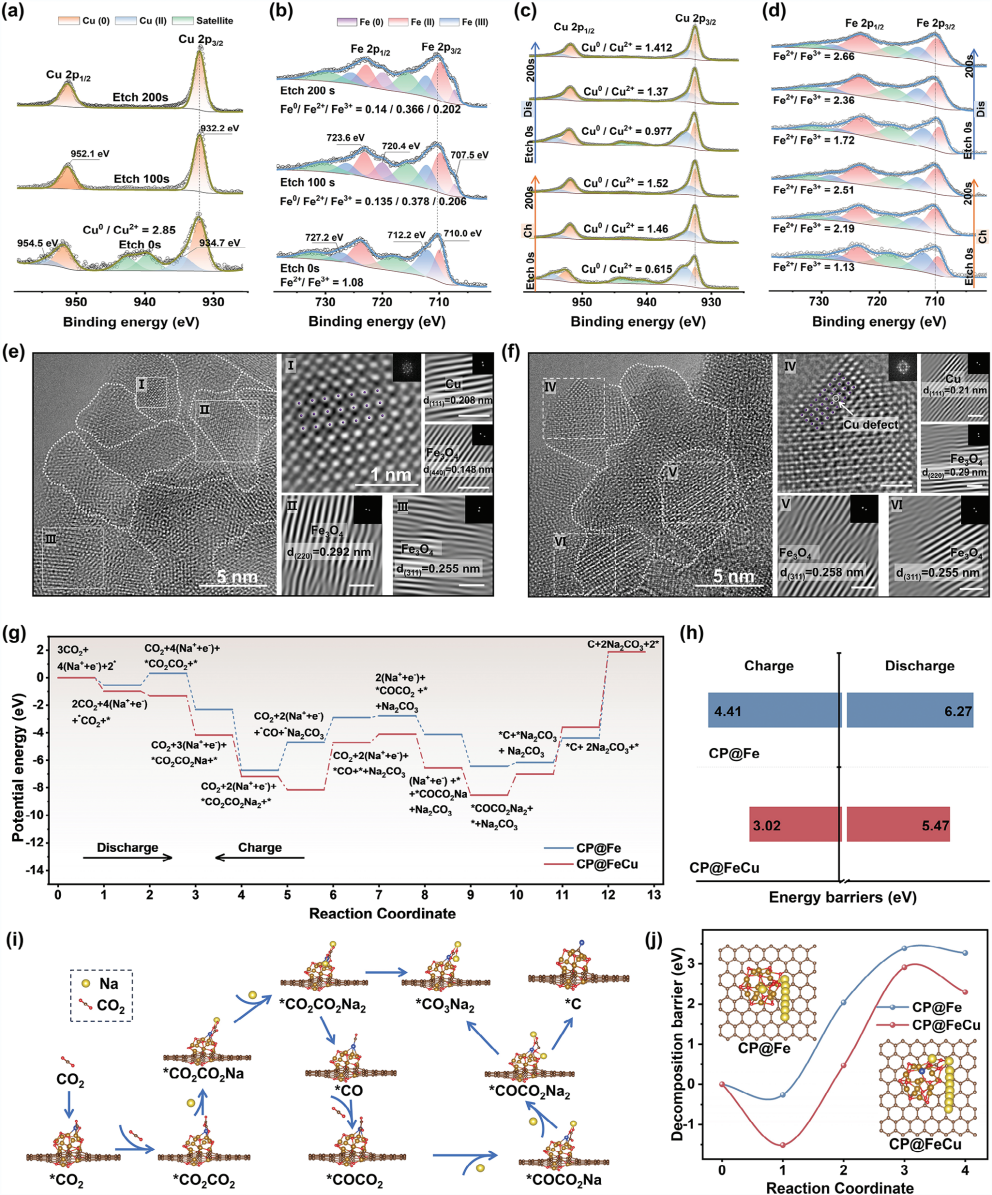
Physicochemical and theoretical investigations of CO_2_RR/CO_2_ER for CP@FeCu. In‐depth XPS spectra of Cu 2p and Fe 2p for CP@FeCu in its a,b) pristine state; c,d) after discharge and recharge. HRTEM images of CP@FeCu after e) discharge and f) recharge. Scale bars: 1 nm. g) Calculated Gibbs free energy differences for the discharge–charge process on CP@Fe and CP@FeCu interface at U = 0 V. h) Corresponding free energy barrier at the rate‐determining step for charge and discharge process. i) The reaction pathways and the optimized structures of intermediates and transition states on the CP@FeCu interface. j) decomposition energy barriers and detailed decomposition paths of Na_2_CO_3_ on CP@Fe and CP@FeCu interfaces.

Inspired by its unique interface structure and favorable composition, CP@FeCu was directly used as a stand‐alone cathode for Na‐CO_2_ batteries. In this case, 1 m NaClO_4_ in TEGDME was chosen as the preferred electrolyte due to its lower volatility than 1 m NaPF_6_ in diglyme. The CP@Fe cathode was also chosen as a control sample. Its electrochemical performance was first evaluated by cyclic voltammetry (CV) at a scan rate of 0.1 mV s^−1^ over a voltage range of 1.8–4.2 V (**Figure** **5**a). The peaks observed at ≈2.3 and 4 V can be attributed to the CO_2_RR and CO_2_ER, respectively. Notably, the CP@FeCu cathode exhibits distinct redox peaks, with a reduction peak at ≈3.17 V and a strong oxidation peak located at ≈3.70 V. In contrast, the CP@Fe cathode does not exhibit significant redox peaks in the 3.17–3.70 V range. The peaks at 3.17 and 3.70 V are likely associated with the valence oscillation of Fe^0^/Fe^2+^/Fe^3+^ and Cu^0^/Cu^2+^, as verified by the subsequent XPS characterizations (Figures 6a–d), indicating that Cu doping promotes charge redistribution between iron and copper species. Valence oscillations not only enhance the performance of CO_2_RR but also play a pivotal role in CO_2_ER, which is indispensable for reducing the overpotential of Na‐CO_2_ batteries.^[^ ^44^, ^45^, ^46^ ^]^ As shown in Figure 5b, the charge‐discharge voltage gap of CP@FeCu cathode‐based Na‐CO_2_ battery stands at 1.55 V, conspicuously lower than the 2.0 V observed in CP@Fe‐based batteries. The deep discharge‐charge characteristics of Na‐CO_2_ batteries featuring CP@FeCu and CP@Fe as cathodes were illustrated in Figure S28 (Supporting Information). The CP@FeCu cathode demonstrates superior performance with higher discharge and charge capacity (≈2120 µAh cm^−2^) compared to the CP@Fe cathode (≈1810 µAh cm^−2^).

The Na‐CO_2_ batteries were further examined for their cycling performance under current densities of 5 and 10 µA cm^−2^ while maintaining a fixed duration of 2 h per cycle (Figures 5c,d). The cycle life of the CP@Fe cathode was only extended to ≈450 h with a stabilized circulation of 210 cycles at 10 µA cm^−2^ due to a restricted number of catalytically active sites promoting CO_2_RR and CO_2_ER. This restriction results in the inability of the discharge product Na_2_CO_3_ to be effectively converted during charging, causing an accumulation of insulated Na_2_CO_3_ during long‐term cycling, which hampers the transport of Na^+^ and CO_2_ and the charge transfer, and finally, thus leading to a notable increase in polarization and eventual battery death during later stages. Remarkably, the CP@FeCu cathode endows the Na‐CO_2_ batteries with a threefold increase in cycling life, reaching 600 stable cycles at 10 µA cm^−2^. Interestingly, the charge‐discharge curves of the CP@FeCu‐based Na‐CO_2_ battery exhibit a low charging voltage with two distinct plateaus during the initial stage of charging (Figure S29, Supporting Information). However, after repeated cycling, these curves evolve into more uniform charge–discharge curves. To further investigate this phenomenon, we analyzed the CV performance of the CP@FeCu‐based Na‐CO_2_ battery over several cycles (Figure S30, Supporting Information). It reveals that the redox peaks associated with the valence state oscillations of Fe and Cu gradually diminish and stabilize after multiple charge‐discharge cycles. This behavior is highly consistent with the charge–discharge curves in Figure S29 (Supporting Information), where, after repeated cycles, only a single plateau remains during charging. This observation indicates that the initial plateau is primarily due to the valence state changes in Fe and Cu, which cause the low initial charging voltage. As the catalyst undergoes repeated cycling, the oxidation states of Fe and Cu reach a more stable configuration (as shown in Figure 6), leading to a more uniform charge–discharge profile. This finding underscores the crucial role that the valence state oscillations of Fe and Cu play in enhancing CO_2_RR and CO_2_ER kinetics, and the stabilization of these redox‐active sites contributes to the enhanced and more stable performance of the battery over prolonged cycling. Furthermore, the battery, utilizing CP@FeCu cathode, demonstrates stable operation at a current density of 5 µA cm^−2^ for 2400 cycles (4800 h) and maintains a high discharge voltage of 2.2 V, which is significantly better than CP@Fe. These results indicate that Cu doping can adjust the electronic structure of CP@Fe, which promotes the activation of CO₂ and the nucleation of Na_2_CO_3_ during discharge while lowering the decomposition barrier of Na_2_CO_3_ during charging, resulting in improved redox kinetics and reduced polarization. More importantly, the exceptional longevity of its cycling notably surpasses previous findings on cycling stability (Figure 5e and Table S2, Supporting Information).^[^ ^2^, ^3^, ^4^, ^5^, ^7^, ^8^, ^10^, ^11^, ^12^, ^13^, ^14^, ^47^, ^48^, ^49^, ^50^, ^51^, ^52^ ^]^ In addition, CP@FeCu‐based Na‐CO_2_ batteries also showed good cycling stability at higher current densities of 50 and 100 µA cm^−2^ (Figure S31, Supporting Information). The above results further indicate that CP@FeCu has excellent catalytic activity and stability for the reversible conversion of CO_2_RR and CO_2_ER processes, which can realize sustainable seasonal energy storage in Na‐CO_2_ batteries.

To better understand the reversible CO_2_RR and CO_2_ER on CP@FeCu cathode, some ex situ measurements including XRD, Raman spectroscopy, and X‐ray photoelectron spectroscopy (XPS), were further carried out to characterize the evolution of the discharge products. Notable changes in the XRD and Raman patterns (Figures 5f,g) of the discharged CP@FeCu cathode are evident, revealing new peaks associated with the formation of Na_2_CO_3_ crystals as the primary discharge products. Upon recharging, all signals associated with Na_2_CO_3_ disappeared, indicating the complete decomposition of Na_2_CO_3_. XPS results further underscore the catalytic activity of CP@FeCu, as evidenced by the appearance and diminution of distinct Na_2_CO_3_ XPS signals at a binding energy of 289.1 eV in the C 1s XPS spectra during full discharge and charge states (Figure S32, Supporting Information). The TEM image in Figure 5h shows that the cathode surface of CP@FeCu is covered with dense products after discharge. The corresponding SAED spectra (Figure 5i) reveal the presence of two distinct phases, where (201), (310), (221), (−512), and (313) diffraction patterns can be indexed to Na_2_CO_3_, while (111) and (311) diffraction patterns are assigned to copper in CP@FeCu. The HAADF‐STEM image and EDS maps, along with the corresponding line‐scan profile of CP@FeCu cathode after discharge, further substantiate that the predominant discharge products exhibit a bulk morphology consisting primarily of Na_2_CO_3_ (Figure S33, Supporting Information). Upon charging, the CP@FeCu cathode surface appears distinctly clear (Figure 5j) and only the diffraction patterns of copper can be seen (Figure 5k), indicating the complete decomposition of Na_2_CO_3_. The evolution of products at the CP@FeCu cathode is demonstrated by SEM images: Na_2_CO_3_ uniformly nucleates and covers the whole carbon fiber surface during discharge (Figure 5l), while in the charging stage, Na_2_CO_3_ undergoes gradual decomposition, ultimately re‐exposing the carbon fiber surface (Figure 5m). This observation strongly supports that CP@FeCu exhibits highly efficient electrocatalytic ability to facilitate the reversible formation and decomposition of Na_2_CO_3_ in Na‐CO_2_ batteries, thereby achieving excellent rechargeability.

To unravel the intrinsic factors contributing to the exceptional catalytic performance of CP@FeCu, in‐depth Cu 2p and Fe 2p XPS analyses were undertaken to elucidate the evolution of the valence states of Fe and Cu from the surface to the bulk within CP@FeCu. For pristine Cu 2p (**Figure** **6**a, bottom), the main peaks at 932.2 and 952.1 eV are attributed to Cu(0) phase, moreover, peaks at 934.7 and 954.5 eV, as well as remarkably pronounced oscillatory satellite peaks at 940.1 and 943.6 eV indicate the presence of Cu(II) phase.^[^ ^53^ ^]^ The disappearance of characteristic peaks associated with Cu(II) as etching time increases suggests the Cu(II) phase is predominantly present on the surface of the CP@FeCu substrate. The pristine Fe 2p spectra (Figure 6b, bottom) reveal two contributions, Fe 2p3/2 and Fe 2p1/2, wherein two pairs of peaks can be discerned. These peaks are further identified as belonging to Fe(II) (710.0 and 723.6 eV) and Fe(III) (712.2 and 727.2 eV).^[^ ^44^ ^]^ The Fe(II)/Fe(III) ratio on the CP@FeCu substrate can be quantified through peak fitting area ratios, indicating approximate equivalence. The appearance of characteristic peaks (707.5 eV) associated with Fe(0) as etching time increases suggests a small amount of Fe(0) phase is present on the bulk of the CP@FeCu substrate. However, the characteristic peaks associated with Fe(0) are shown on the surface of the CP@Fe substrate (Figure S34, Supporting Information). In addition, the binding energy of Fe 2p in CP@FeCu shifts to a higher binding energy with an increased proportion of Fe in the oxidized state compared to CP@Fe. This phenomenon arises from charge transfer from Fe to Cu atoms, which is consistent with charge depletion around Fe and charge accumulation around Cu in the charge density difference (Figure 1c). The in‐depth XPS survey image of CP@FeCu (Figure S35, Supporting Information) reveals an increase in the number of copper species with increasing leaching time, while nitrogen species are observed only on the surface of the CP@FeCu substrate. Compared to the initial sample, after the discharge and charging process (Figures 6c,d), the disappearance of the characteristic signal of Fe^0^ and the significant enhancement of the signals of Fe^3+^ and Cu^2+^ are observed. This clarifies that the charge redistribution on these two elements has an important role in CO_2_RR/CO_2_ER. Quantitative analysis reveals that the redox states of Fe and Cu oscillate from the surface to the bulk during discharge and charging. These results indicate that the valence oscillation of Fe and Cu sites induced by charge redistribution promotes remarkably high CO_2_RR/CO_2_ER activity.

The multiscale defect structures of CP@FeCu samples after the discharged and charged states were further investigated. HRTEM images (Figures 6e,f) reveal that the interconnected structure of nanoparticles with abundant grain boundaries consists of numerous crystalline‐amorphous nanodomains. Enlarged HRTEM images and corresponding IFFT images confirm that both discharged and charged CP@FeCu maintain defective structural features, such as Cu atom defect, edge dislocations, and distinctly distorted atomic arrangements. Additionally, it is observed that Cu atoms in the Cu‐rich regions penetrate and extend into the lattice of Fe_3_O_4_ grains (the enlarged image of Region I in Figure 6e, and the enlarged image of Region VI in Figure 6f. This penetration leads to complex lattice distortions and nanostructural defects, which inevitably enhance the dynamics of the electron distribution or oxidation state between the defective Fe─Cu active sites, thereby promoting the catalytic process of CO_2_RR/CO_2_ER. Well‐preserved structures of various defects are still observed within CP@FeCu after charging and discharging cycles, indicating the high stability of multiscale defect interfaces. Such defects maintain high structural durability and functional stability, thereby ensuring excellent cyclic stability of CP@FeCu in prolonged cycles.

To elucidate the intrinsic connection between the CP@FeCu interface with optimized electronic structure and the overall enhanced catalytic activity in the Na‐CO_2_ conversion, the Gibbs free energy evolution for each step of the charge/discharge process at U = 0 V was calculated (Figure 6g). Based on the four‐electron reaction pathway,^[^ ^2^, ^54^, ^55^ ^]^ it can generally be assumed that CO_2_ER is an inverse reaction of CO_2_RR, implying that these two reactions share identical active sites and reaction intermediates. Figure 6i and Figure S36 (Supporting Information) depict possible reaction pathways at the interfaces of CP@FeCu and CP@Fe, along with optimized structures of intermediates and transition states. As illustrated in Figure 6h, the discharge process shows a maximum free energy barrier (ΔG_max_) of 6.27 eV for CP@Fe and 5.47 eV for CP@FeCu interface. While, ΔG_max_ values of 4.41 and 3.02 eV were obtained for CP@Fe and CP@FeCu interfaces respectively, during the charging process. These findings suggest that CO_2_RR and CO_2_ER are more thermodynamically favorable on the CP@FeCu interface due to significantly lower free energy barriers compared to those on the CP@Fe interface. In addition, it is known from initial theoretical analyses that the charge redistribution between Fe and Cu atoms can weaken the interactions between the Na─O bonds, leading to a favorable decomposition of Na_2_CO_3_. Further, the decomposition barriers of Na_2_CO_3_ at these two interfaces were also calculated (Figure 6j). A decreased decomposition barrier of Na_2_CO_3_ can significantly enhance the efficiency of active site utilization, reduce the formation of dead Na_2_CO_3_, and lower the charging voltage. The decomposition barrier of Na_2_CO_3_ on the CP@FeCu interface, is significantly smaller than that on the CP@Fe surface, which is consistent with experimental findings. Overall, the adsorption behaviors, potential interfacial electronic structures, and property relationships of CP@FeCu have been investigated based on the simplified FeCu cluster model with under‐coordinated atoms, which provides a theoretical explanation for the remarkable catalytic behavior of defective active FeCu species within CP@FeCu. Nevertheless, incorporating defect structures (e.g., vacancies, grain boundaries, dislocations, etc.) shall contribute to a deeper understanding of the relationship between multiscale defects and properties in future research.

### 2.5 Investigating the Feasibility of Anode‐Less Na‐CO_2_ Batteries

The above experimental and theoretical calculations demonstrate that multiscale defective FeCu interfaces play an important role in improving the performance of Na deposition and Na‐CO_2_ conversion. Specifically, the addition of Cu into CP@Fe introduces a variety of defect sites, causing distortions in the lattice structure and altering the distribution of charges at the interface. Meanwhile, the upward shift of the d‐band center enhances the ability of reactants to bind at the CP@FeCu interface and promotes their effective activation. For Na deposition, the electron‐rich state surrounding the FeCu atoms optimizes electron transfer across the carbon skeleton interface and promotes rapid migration of Na^+^ ions. This increases Na^+^ binding energy and reduces overpotential for Na‐nucleation, enabling uniform distribution of Na^+^ throughout the carbon skeleton rather than top growth. For Na‐CO_2_ conversion, the coupling of multiscale defects and electron‐rich FeCu environments maximizes the excitation of active sites, thereby enhancing CO_2_ activation and Na_2_CO_3_ nucleation during the discharge process. Additionally, it weakens the interaction of Na─O bonds to reduce the decomposition barrier of Na_2_CO_3_ during charging, ultimately improving the reaction kinetics of CO_2_ER and CO_2_RR.

**Figure 7:**
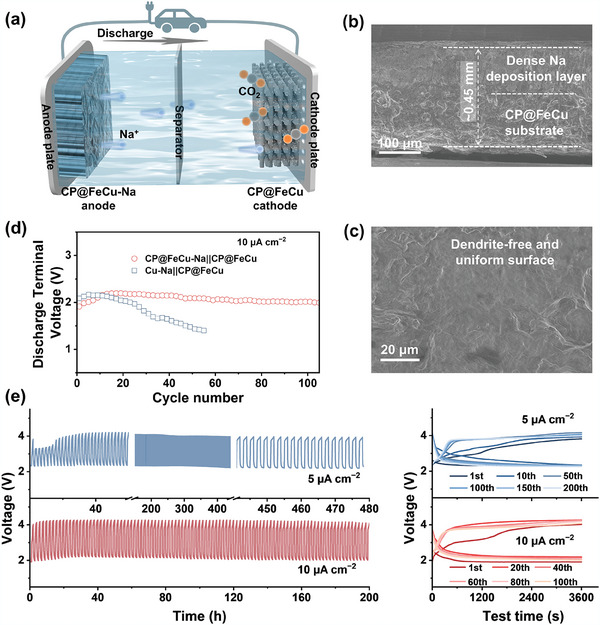
a) Schematic of the cell configurations for anode‐less Na‐CO_2_ batteries employing CP@FeCu as both Na anode host and CO_2_ cathode. b,c) SEM cross‐sectional and top‐sectional images of Na metal deposited on CP@FeCu substrate at a capacity of 50 mA h cm^−2^. d) Comparison of discharge terminal voltages of CP@FeCu‐Na||CP@FeCu and Cu‐Na||CP@FeCu cells at 10 µA cm^−2^. e) Long‐term cycling performance and selected charge–discharge curves for CP@FeCu‐Na||CP@FeCu cells at 5 and 10 µA cm^−2^.

With this in mind, to assess the feasibility of CP@FeCu bifunctional electrodes for practical applications, proof‐of‐concept for anode‐less Na‐CO_2_ batteries solely using CP@FeCu was also investigated. The comparative analysis was conducted between a full‐cell configuration utilizing CP@FeCu‐Na anode and CP@FeCu cathode (CP@FeCu‐Na||CP@FeCu) (**Figure** **7**a). As shown in Figures 7b,c, the CP@FeCu‐Na electrode maintains dense and smooth deposition morphology with an overall electrode thickness of ≈0.45 mm and a sodium loading of 50 mAh cm^−2^. In contrast, an uneven surface with Na dendrites is seen in the sodium deposition pattern on the copper foil when an identical amount of sodium was deposited (Figure S37, Supporting Information). Figure S38 presents the CV performance of the CP@FeCu‐Na||CP@FeCu and CP@Fe‐Na||CP@Fe cells at a scan rate of 0.1 mV s^−1^, which closely matches the CV performance of the pure Na‐CO_2_ battery containing CP@FeCu and CP@Fe (Figure 5a). This strong agreement suggests that both cells follow the same reaction mechanism, and further verifies the decisive influence of Cu doping‐induced valence oscillation on catalytic activity of CP@FeCu. Unsurprisingly, there are notable variations in the full battery based on these two anodes. As demonstrated in Figure 7d, the terminal discharge voltage of CP@FeCu‐Na||CP@FeCu cell remains greater than 2 V after 100 cycles, while the Cu‐Na||CP@FeCu cell declines rapidly after only 20 cycles. Moreover, the CP@FeCu‐Na||CP@FeCu cells demonstrate exceptional cycling stability, enduring over 240 cycles (480 h) at 5 µA cm^−2^ and exceeding 100 cycles (200 h) at 10 µA cm^−2^, both while maintaining nearly constant charge/discharge curves (Figure 7e). Conversely, at the same current density, the charge/discharge voltage of the full cell composed of bare Na (Cu‐Na||CP@FeCu) drops rapidly after 40 h of cycling (Figure S39, Supporting Information). This difference can be attributed to the instability of the Cu‐Na anode during the plating/stripping process, while the CP@FeCu‐Na anode is more resilient. Overall, CP@FeCu not only serves as a 3D host for Na deposition but also prevents Na ion loss due to its excellent reversibility. Even in an open environment such as Na‐CO_2_ cells, a very low sodium metal usage can significantly improve the cycle life of Na‐CO_2_ batteries. Therefore, this work is useful for the future preparation of high‐energy‐density anode‐less and anode‐free sodium metal batteries, including but not limited to Na‐CO_2_ batteries.

## 3 Conclusions

In summary, we have demonstrated a CP@FeCu “two‐in‐one” electrode with multiscale defective interfaces can serve as the sodium metal host and CO_2_‐breathing cathode targeting long‐life Na‐CO_2_ batteries. Microstructural, electrochemical reaction, and theoretical calculations investigations collectively reveal the relationship between multiscale FeCu defective structure and performance for sodium deposition and CO_2_ redox behavior in Na‐CO_2_ batteries. It is found that a CP@FeCu electrode with sodiophilic nature would induce homogeneous growth of Na to effectively reduce the nucleation overpotential and suppress the dendritic formation. In addition, such a multiscale FeCu defective structure is also able to work as an effective catalyst for accelerating CO_2_ conversion owing to synergistic and valence oscillation effects. Consequently, the CP@FeCu electrode exhibits excellent bidirectional electrochemical performance. Serving as a sodiophilic host, it enables the uniform nucleation and stable cycling of sodium with a low overpotential for Na‐nucleation. And, functioning as a CO_2_ cathode in Na‐CO_2_ batteries, it displays excellent cycling stability of 2400 cycles (4800 h) at 5 µA cm^−2^. Furthermore, anode‐less Na‐CO_2_ batteries exhibit long‐duration sustainability. The findings in this work may shed new light on the systematic design of “two‐in‐one” electrodes based on the superiority of multiscale defective interfaces for energy conversion and storage fields and beyond.

## Conflict of Interest

The authors declare no conflict of interest.

## Supporting information



Supporting Information

Supplemental Movie 1

Supplemental Movie 2

Supplemental Movie 3

## Data Availability

The data that support the findings of this study are available in the supplementary material of this article.
