# Supplementary material for: Multiscale Defective Interfaces for Realizing Na‐CO2 Batteries With Ultralong Lifespan
Source: Adv Mater. 2024 Oct 9;36(48):2409533. doi: 10.1002/adma.202409533 (PMC11602679; doi:10.1002/adma.202409533)
Supplement: Supplementary file 1 — Supporting Information [file ADMA-36-2409533-s002.docx]

# Multiscale Defective Interfaces for Realizing Na-CO_2_ Batteries with Ultralong Lifespan

*Changfan Xu^1^, Ping Hong^1^, Yulian Dong^1^, Yueliang Li^2^, Yonglong Shen^3^, Johannes Biskupek^2^, Huaping Zhao^1^, Ute Kaiser^2^, Guosheng Shao^3,^*, and Yong Lei^1,^**

^1^Fachgebiet Angewandte Nanophysik, Institut für Physik & IMN MacroNano, Technische Universität Ilmenau, 98693 Ilmenau, Germany

Email: [yong.lei@tu-ilmenau.de](mailto:yong.lei@tu-ilmenau.de)

*^2^*Central Facility for Electron Microscopy, Electron Microscopy Group of Materials Science, Ulm University, Ulm, 89081, Germany

^3^School of Materials Science and Engineering, Zhengzhou University, Zhengzhou 450001, China

Email: gsshao@zzu.edu.cn

## Experimental Section

*Materials and chemicals:* Potassium ferricyanide(III) (K_3_[Fe(CN)_6_], AR, ≥99.0%), Iron(III) chloride hexahydrate (FeCl_3_·6H_2_O, AR, 99.0-102.0%), ethanol (96%, v/v) were sourced from VWR Chemicals. Sodium (Na, ≥99.9%), tri-Sodium citrate dihydrate (Na_3_C_6_H_5_O_7_·2H_2_O, AR, ≥99%) was ordered from Merck chemical. Copper(II) acetate (Cu_2_(CH_3_COO)_4_, AR, 98.0-102.0%) and Sodium perchlorate, anhydrous (NaClO_4_, AR, 98.0-102.0%) were purchased from Alfa Aesar. Sodium hexafluorophosphate (NaPF_6_, AR, ≥99%), Diethylene glycol dimethyl ether (diglyme, AR, 99%), Tetraethylene glycol dimethyl ether (TEGDME, AR, 99%), Toray carbon paper (CP, TGP-H-60) was purchased from Thermo Scientific. All chemicals in this study were used directly without further purification.

*Synthesis of CP@FeCu*: 0.4 g of Cu_2_(CH_3_COO)_4_ and 1.4 g of Na_3_C_6_H_5_O_7_·2H_2_O were solved in 30 mL of ethanol and deionized (DI) water (v/v 1:1). Toray Carbon paper was then immersed in the above solution. Another mixture of the same solvents containing 0.48 g of K_3_[Fe(CN)_6_] was slowly added to the above solution with stirring. 2 hours later, the resulting green mixture was incubated at room temperature for 72 hours. The obtained carbon paper with red precipitates was washed with DI water and ethanol, and dried at 60 °C overnight. Following this, the obtained precursor carbon paper was pyrolyzed at 800 °C under nitrogen at a ramp rate of 1 °C min^−1^. Finally, the prepared samples were washed by immersion in deionized water and dried at 60 °C overnight to obtain CP@FeCu.

*Synthesis of CP@Fe*: 0.54 g FeCl_3_·6H_2_O was first dissolved in a solvent mixture of 15 mL ethanol and 15 mL DI water to form a yellow-brown solution and 0.33 g K_3_Fe(CN)_6_  was dissolved in a separate identical solvent mixture to form a light-yellow solution. Toray Carbon paper was soaked in K_3_Fe(CN)_6_ solution, and then the FeCl_3_ solutions were dropwise added into it under stirring. After 2 hours, the mixed solution was aged for 72 hours at room temperature. The obtained dark blue carbon paper was washed with DI water and ethanol, and dried at 60 °C overnight. Subsequently, CP@Fe was obtained by following the same procedure as for CP@FeCu.

*Physicochemical Characterizations*: The X-ray diffraction (XRD) patterns were collected using a SIEMENS D5000 diffractometer equipped with Cu Kα radiation (λ = 0.15406 nm). Scanning electron microscopy (SEM) images were captured through ZEISS AURIGA 60 field emission scanning electron microscope. High-resolution (HR) transmission electron microscopy (TEM) and selected area electron diffraction (SAED) images were captured using the Cc/Cs-corrected Sub-Angström low-voltage electron microscope (SALVE)^[1]^ with an acceleration voltage of 80 kV. Scanning transmission electron microscopy (STEM) coupled with energy-dispersive X-ray (EDX) analysis was performed using a TEM a Thermo Fisher Talos operated at 200 kV. Raman spectra were recorded using NT-MDT Spectrum Instruments with a 532-nm laser. X-ray photoelectron spectroscopy (XPS, Thermo K-alpha) was used to characterize the materials’ detailed chemical composition and chemical valence states. Electron paramagnetic resonance (EPR, Bruker EMXplus-6/1) spectra was used to analyze the concentration of oxygen vacancies. The investigation of sodium ion plating behavior on the target electrode was conducted utilizing Nikon optical microscopes.

*Electrochemical Characterizations:* All electrochemical assessments employed in this research were assembled using 2032-type coin cells, and all components of the cells and in situ dendritic test mold were procured from Guangdong Canrd New Energy Technology Co.Ltd. A glovebox filled with nitrogen (N_2_) containing water (H_2_O) and oxygen (O_2_) concentrations below 0.1 parts per million (ppm) was employed for the assembly of various cells. The electrochemical cycling procedures were carried out at 25 °C utilizing a Land CT 2001A battery testing apparatus from Wuhan Land Electronics Co., Ltd., China. Cyclic voltammetry (CV) experiments were performed using a VSP electrochemical workstation (Bio-Logic, France).

*Electrochemical* *Testing of Na Plating/Stripping:* The half cells were composed of Na foils serving as the counter/reference electrode (Φ= 10 mm), two layers of Celgard 2400 as the separator, 1 M NaPF_6_ in diglyme as the electrolyte, and the working electrodes, including CP@FeCu, CP@Fe, CP, and Cu foil. These working electrodes were used directly without the addition of conductive agents and binders. To assess cycling stability, the cells underwent an initial operation for 5 cycles at a galvanostatic current of 0.1 mA cm⁻², ranging between 0.01 and 1 V (vs. Na/Na^+^), aiming to eliminate contaminants and establish a stable solid electrolyte interphase (SEI). The Coulombic efficiency (CE) for each cycle was determined by calculating the ratio of the total stripping capacity to the total deposition capacity. For the Aurbach CE test,^[2]^ the half-cells underwent a pre-activation phase, where 5 mAh cm^−2^ of sodium was deposited on the prepared electrodes and stripped to 1 V in the initial cycle to stabilize the electrode surfaces. Subsequently, an additional 5 mAh cm^−2^ of sodium was deposited again on the electrode as a Na reservoir, followed by 10 cycles of Na stripping/plating for 1 mAh cm^−2^. Finally, the remaining Na was stripped to 1.0 V. The Aurbach CE calculation can be based on the following equation:

$$\text{CE}_{\text{Avg.}}\text{ }\left( \text{\%} \right)\text{=100×}\frac{\text{n}\text{Q}_{\text{c}}\text{+}\text{Q}_{\text{s}}}{\text{n}\text{Q}_{\text{c}}\text{+}\text{Q}_{\text{d}}}$$

where *n* represents the cycle number (10 cycles), *Q_c_* is the Na plating and stripping capacity in each cycle (1 mAh cm^−2^), *Q_d_* is the initial Na plating capacity (5 mAh cm^−2^), and *Q_s_* is the final stripped Na capacity after 10 stripping/plating cycles.

*Electrochemical Testing of Na-CO_2_ batteries:* Na-CO_2_ batteries were assembled, featuring an excess sodium foil as the anode, 1 M NaClO_4_ in TEGDME as the electrolyte, and a glass fiber membrane (Whatman, GF/B) serving as the separator. The freestanding CP@FeCu and CP@Fe were directly employed as CO_2_-breathing cathodes, and the cathode shell was designed with holes to enable CO_2_ access to the electrode. The assembled batteries were placed in a pure CO_2_-filled metal-air coin cell test chamber (NJ Scientific Ltd., China). Before performing galvanostatic testing at room temperature, the battery was first left to stand in a CO_2_ environment for 2 hours.

*Assembly and electrochemical testing of anode-less Na-CO_2_ batteries:* The Na-deposited CP@FeCu is used as the anode (CP@FeCu-Na) and CP@FeCu as the cathode to build the anode-less Na-CO_2_ batteries with CP@FeCu-Na||CP@FeCu. To make CP@FeCu-Na electrodes and to control the amount of sodium, 50 mAh cm^−2^ of Na was plated onto CP@FeCu electrodes in a half-cell at a current density of 1 mA cm^−2^, and the mass of sodium was ~34 mg. The prepared CP@FeCu-Na was then extracted from the half-cell and cleaned with TEGDME for future use. Anode-less Na-CO_2_ batteries based on Cu-Na||CP@FeCu were also prepared for comparison in a similar manner.

*Theoretical calculations:* Given these structural characteristics of CP@FeCu, a simplified model of a Fe_3_O_4_ cluster doped with a single copper atom is rationalized for the construction of catalytically active FeCu species to study their potential interfacial electronic structure and property relationships. The spin calculations were conducted utilizing the Vienna ab initio simulation package (VASP), employing the projector-augmented plane-wave method within the density functional theory (DFT) framework.^[3, 4]^ The selection of the exchange-correlation potential utilized in the generalized gradient approximation (GGA-PBE) was based on the suggestion put forth by Perdew, Burke, and Ernzerhof.^[5, 6]^. The adsorption energy (E_ads_) was calculated using the formula: E_ads_=E_surface+adsorbate_−(E_surface_+E_adsorbate_), where: E_surface+adsorbate_ is the total energy of the FeOCu cluster with the adsorbates (Na, CO_2_, or Na_2_CO_3_) present, E_surface_ is the energy of the clean FeOCu surface, E_adsorbate_ is the energy of the isolated adsorbate in its reference state. A cut-off energy of 400 eV was selected for the plane wave, while the iterative solution of the Kohn-Sham equation employed an energy criterion of 1×10^−6^ eV.

*COMSOL Simulation:* In the simplified two-dimensional model, the COMSOL Multiphysics 5.6 Finite Element Simulation was utilized to simulate the electric field, current density, and Na ion concentration distribution. The electric field is simulated by using the current module in the built-in AC/DC module of COMSOL. The morphological change and electrochemical current distribution of the sodium plating process were calculated by transient simulation by coupling the current distribution module and the phase field module. The electrochemical processes occurring at the electrode surface are governed by the Butler-Volmer equation, while the concentration diffusion of ions adheres to Fick's law. Simultaneously, the electric migration of ions complies with the Nernst-Einstein relation. The three models used in the experiments are: unloaded bare carbon fiber, carbon fiber loaded with iron oxide nanoparticles, and carbon fiber loaded with copper-doped iron oxide nanoparticles. To demonstrate the role of loaded iron oxide nanoparticles and copper-doped iron oxide nanoparticles, a high-mobility layer was applied to the particle surface to account for the impact of uniform ionic flux and current distribution.

## Supporting Figures and Tables


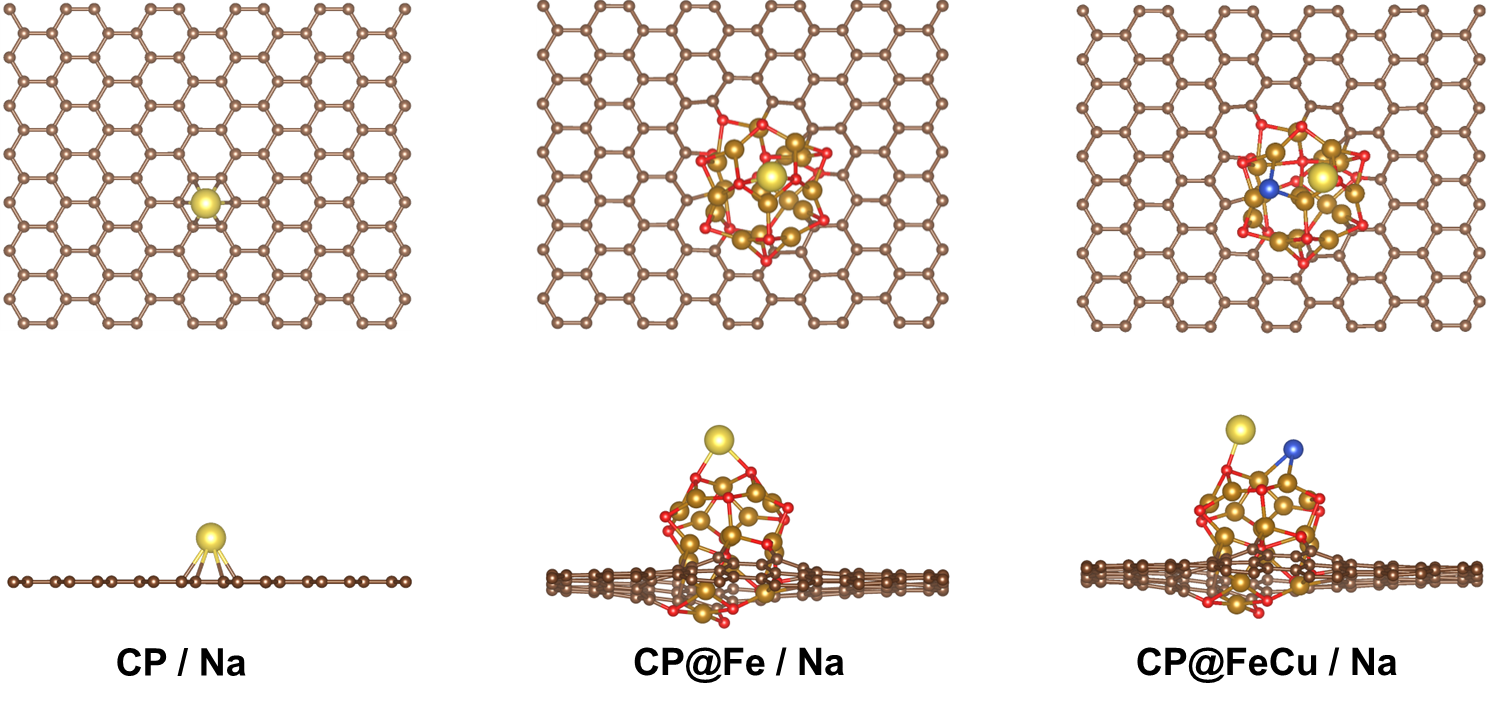


Figure S1. Top and side views for the Na adsorption configurations on CP, CP@Fe, and CP@FeCu, respectively.


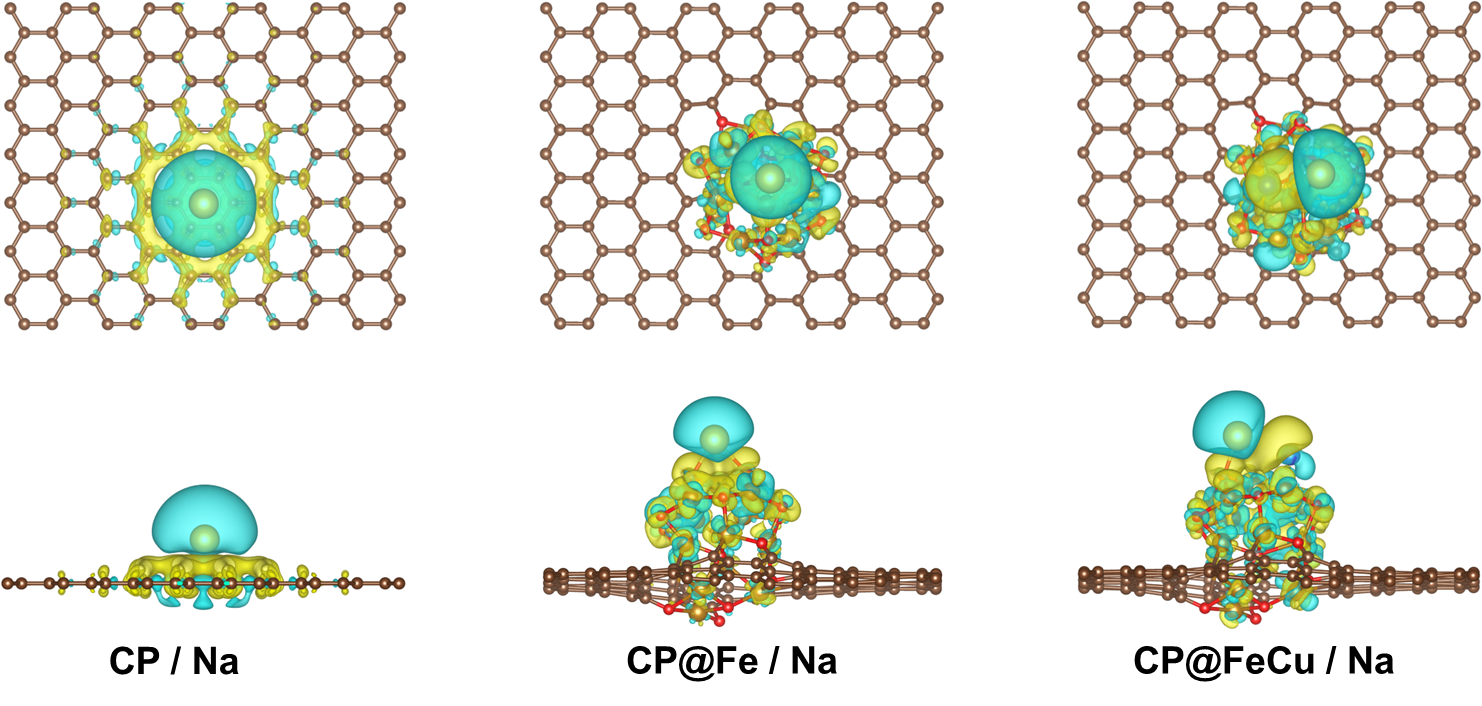


Figure S2. Top and side views for the charge density difference of Na adsorption configurations on CP, CP@Fe, and CP@FeCu, respectively.


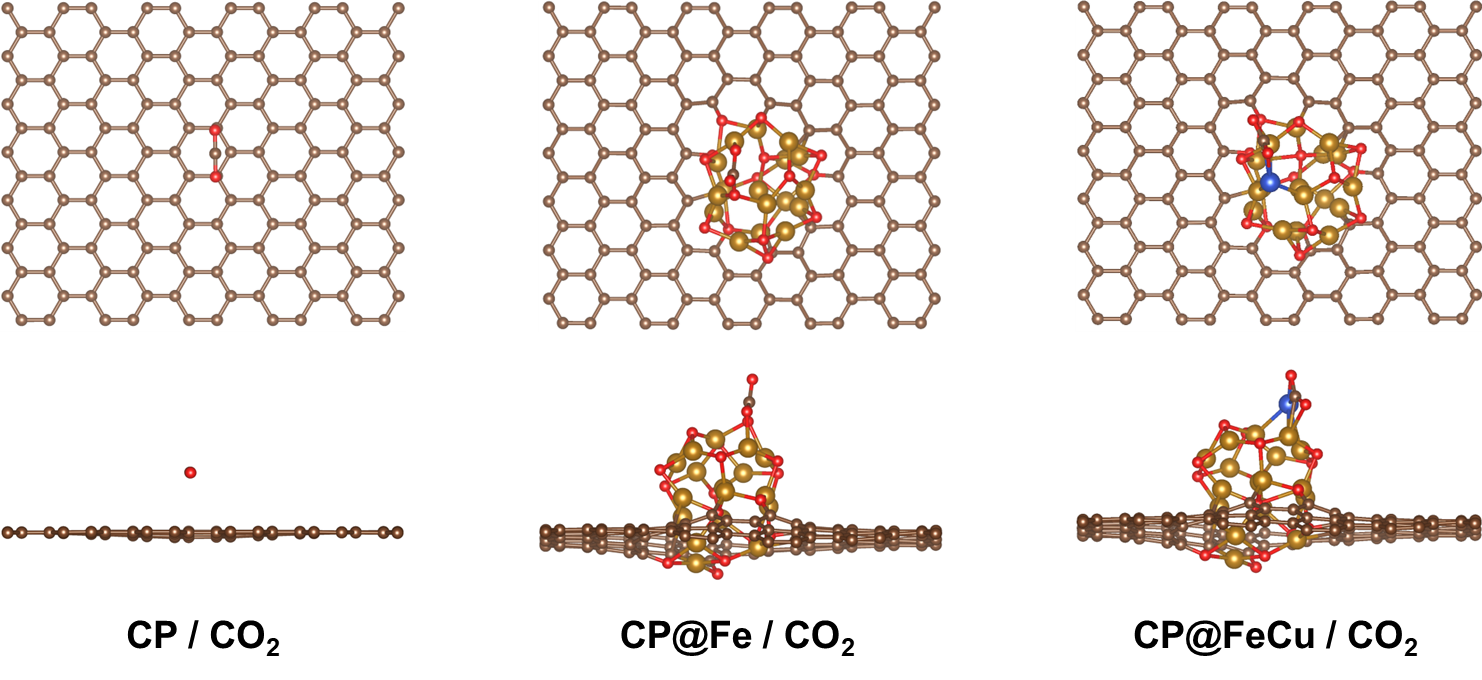


Figure S3. Top and side views for the CO_2_ adsorption configurations on CP, CP@Fe, and CP@FeCu, respectively.


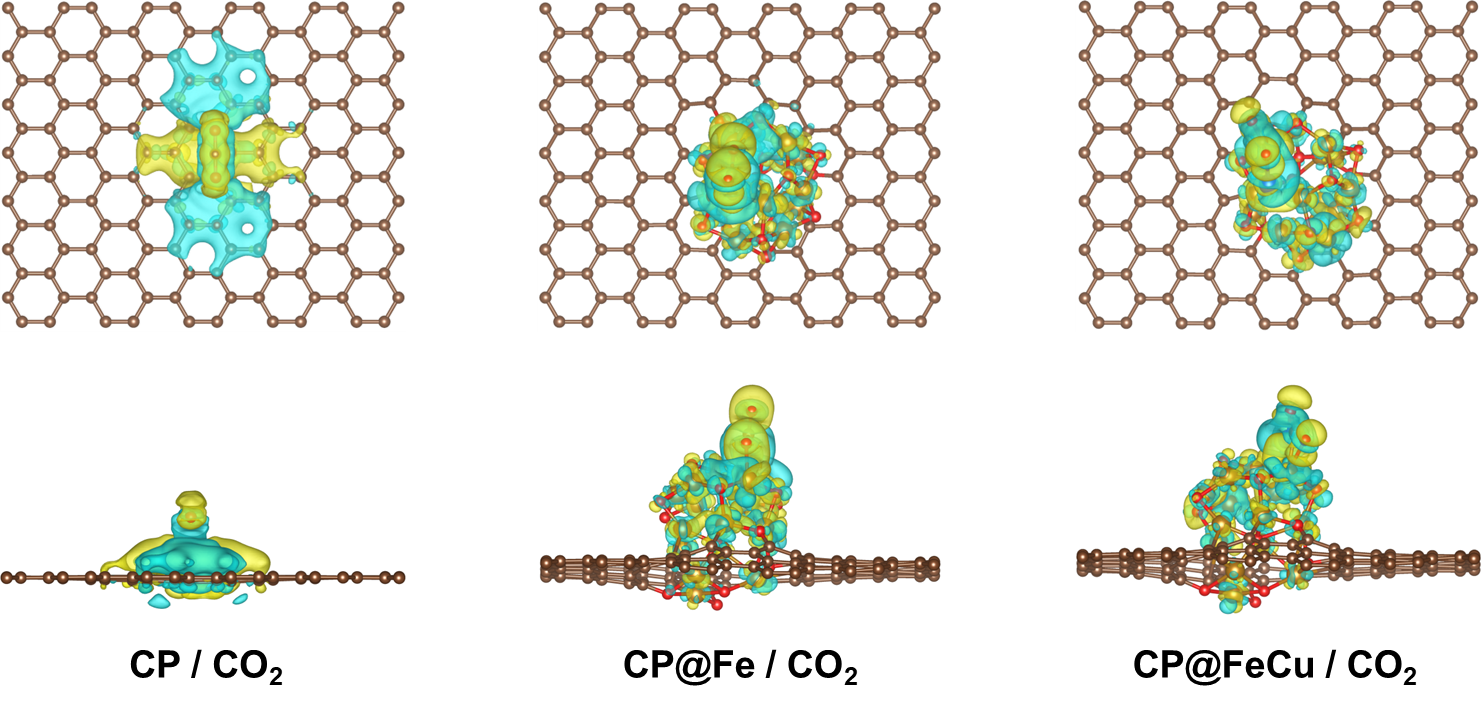


Figure S4. Top and side views for the charge density difference of CO_2_ adsorption configurations on CP, CP@Fe, and CP@FeCu, respectively.


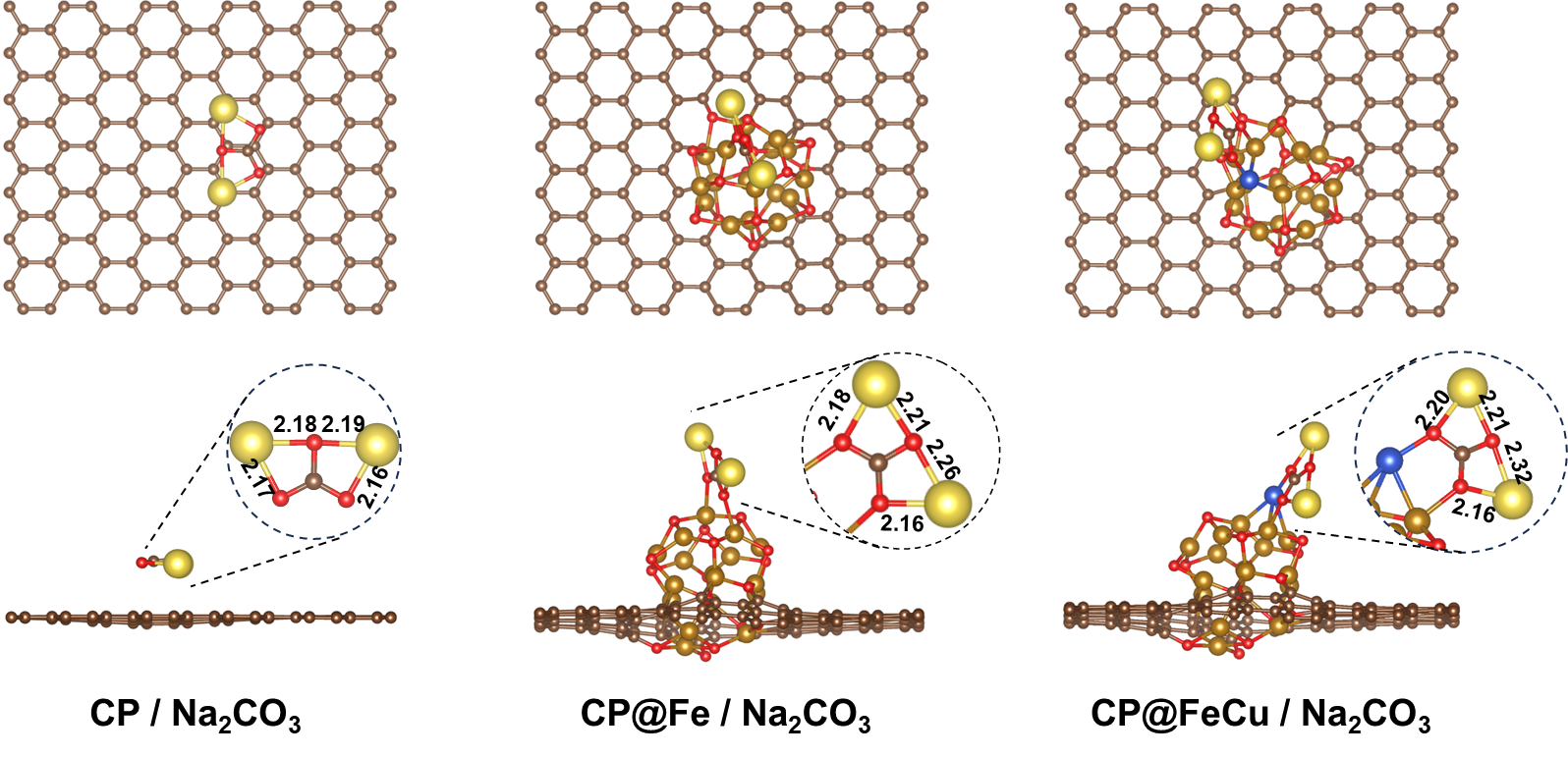


Figure S5. Top and side views for the Na_2_CO_3_ adsorption configurations on CP, CP@Fe, and CP@FeCu, respectively.


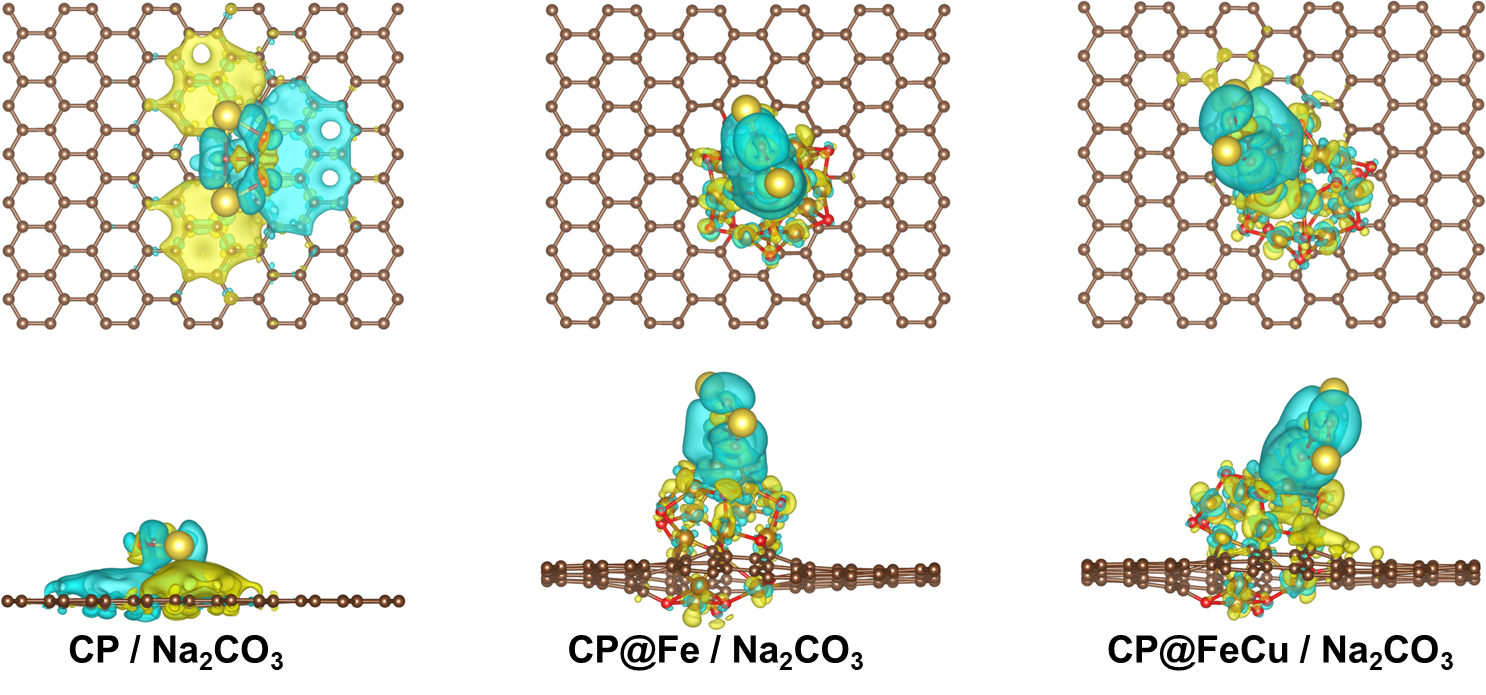


Figure S6. Top and side views for the charge density difference of CO_2_ adsorption configurations on CP, CP@Fe, and CP@FeCu, respectively.


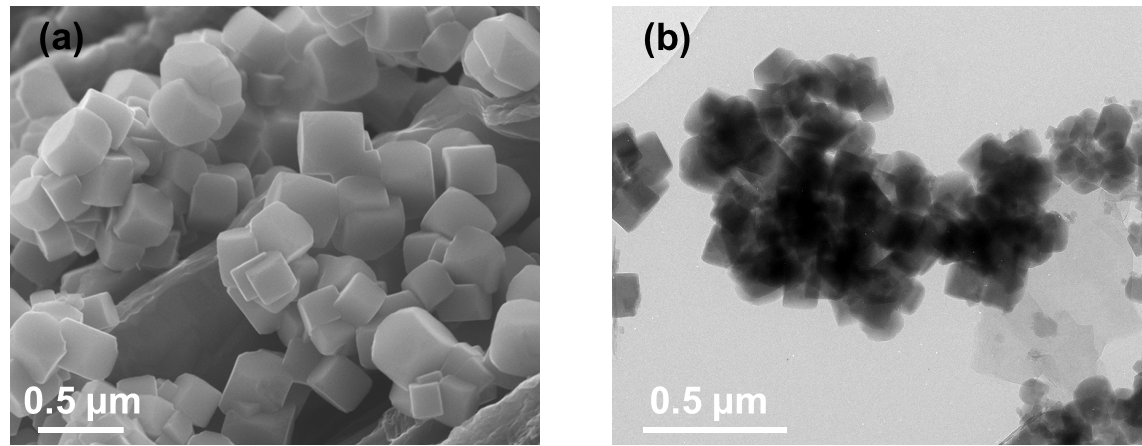


Figure S7. (a) SEM and (b) TEM images of CP@FeCu Prussian blue analogues


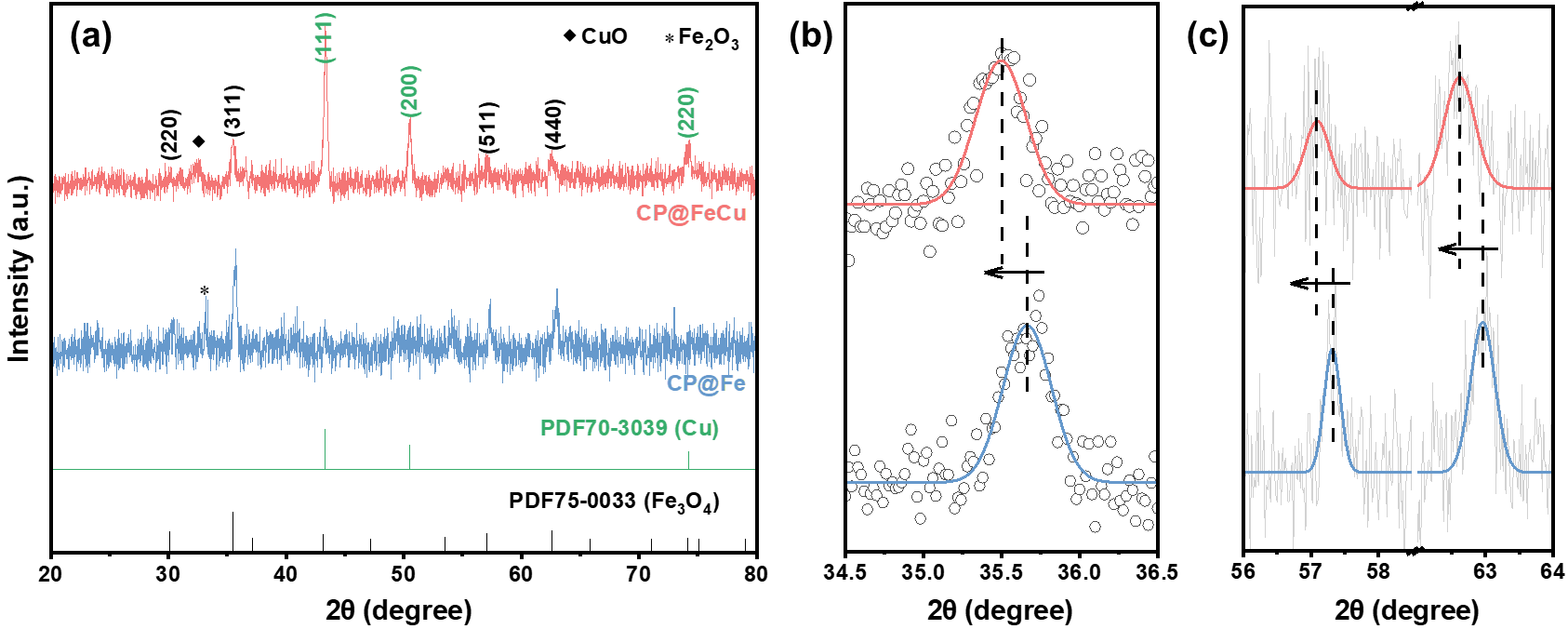


Figure S8. (a) powder XRD patterns of FeO*_x_* and Cu-FeO*_x_* for CP@Fe and CP@FeCu electrodes. (b, c) The partial magnification and profile fitting of the corresponding diffraction peaks in XRD patterns for the (311), (511), and (440) facets.


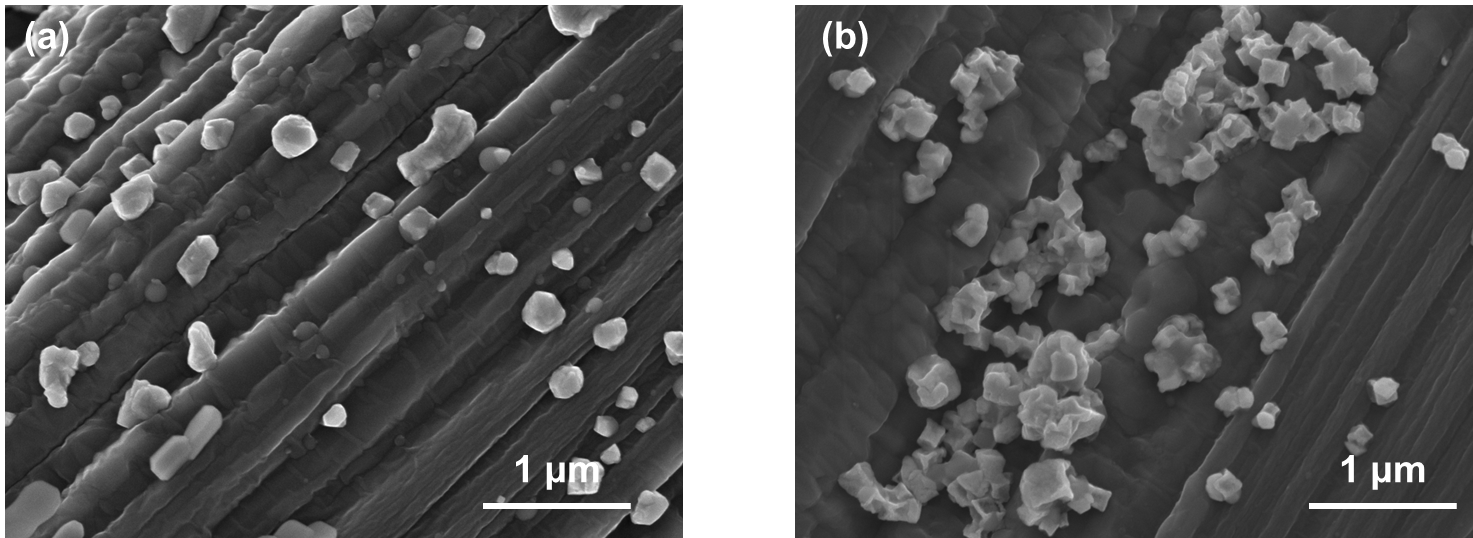


Figure S9 SEM images of (a) CP@Fe and (b) CP@FeCu


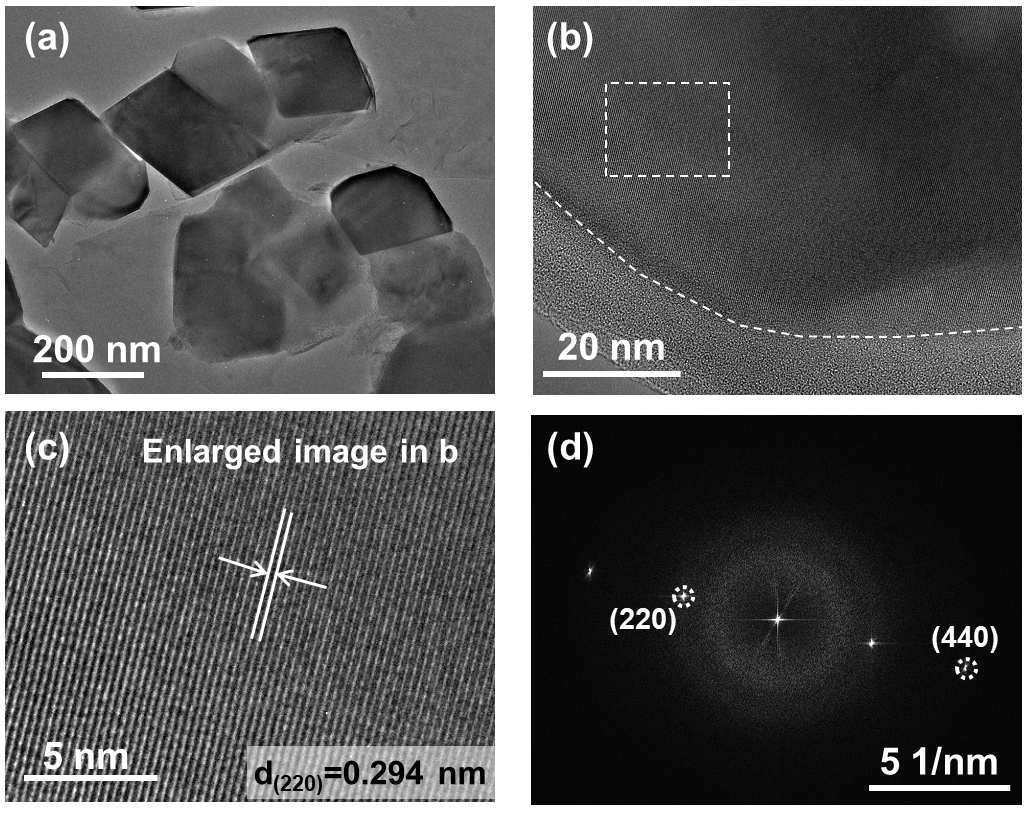


Figure S10. TEM images of the CP@Fe samples. (a) TEM images of the CP@Fe, (b, c) HRTEM images of the CP@Fe samples, and (d) corresponding FFT pattern.


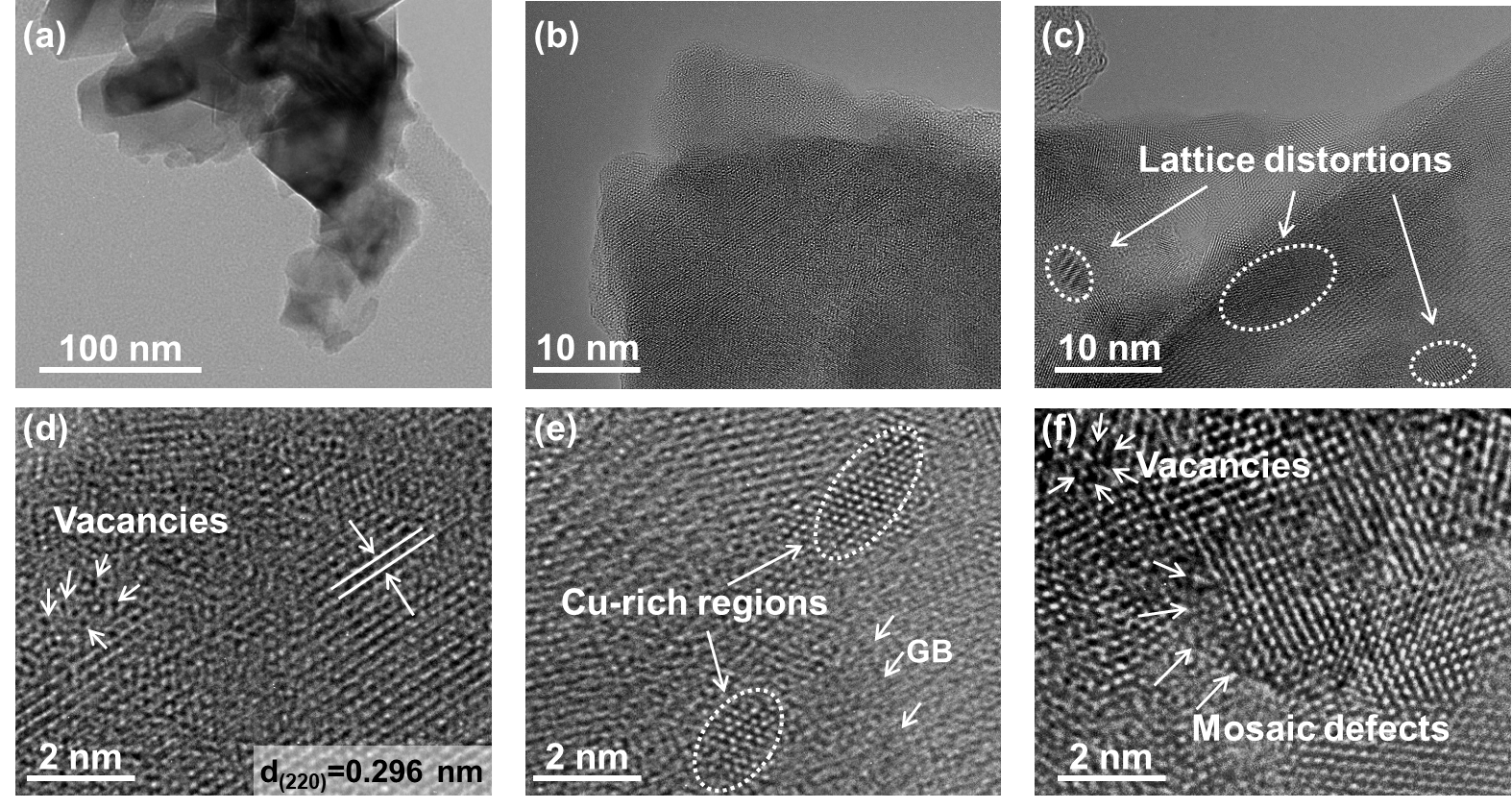


Figure S11. TEM images of the CP@FeCu samples. (a) TEM images of the CP@FeCu, (b, c) HRTEM images of the CP@FeCu samples, (d-f) Additional TEM images of the CP@FeCu samples taken from 3 random-selected areas.


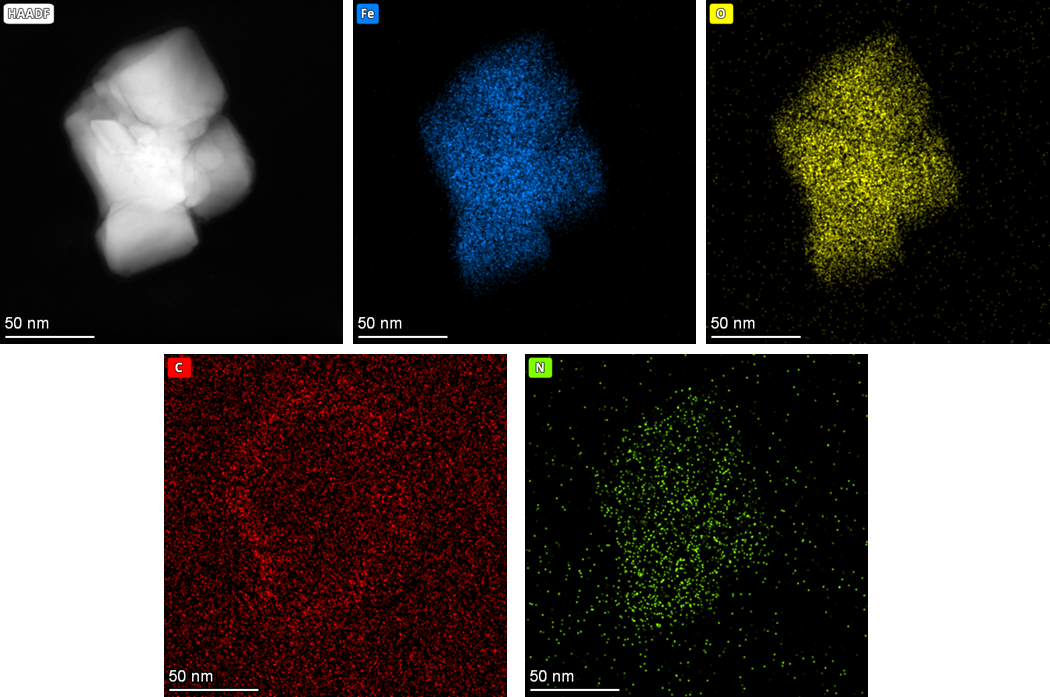


Figure S12. HAADF-STEM image and EDS maps of CP@Fe.

Figure S13. Voltage profiles of Na nucleation at a current density of 1 mA cm^−2^.

Figure S14. Galvanostatic profiles of the initial activation process for CP, CP@Fe, and CP@FeCu.

Figure S15. Galvanostatic profiles of the initial activation process for Cu foil.

Figure S16. Galvanostatic profiles of the final cycles for different hosts.

Figure S17. Aurbach CE tests for calculating average coulombic efficiencies for CP, CP@Fe, and CP@FeCu.

Figure S18. Rate performances at various areal current densities.

Figure S19. CV curves of asymmetric Na cells with CP, CP@Fe, and CP@FeCu.

Figure S20. Nyquist plots of the asymmetric cells with CP, CP@Fe, and CP@FeCu electrodes, and corresponding equivalent circuit.

Figure S21. ESR spectra of CP@Fe, and CP@FeCu.

Figure S22. Special cycling stabilities of the Na metal anodes. Initially, 5 mAh cm^−2^ of Na metal was plated onto the substrates, followed by reversible plating/stripping cycles at 1 mAh cm^−2^ and 1 mA cm^−2^_._ The inset shows the zoomed-in image for the cycling time of 0~15 hours.


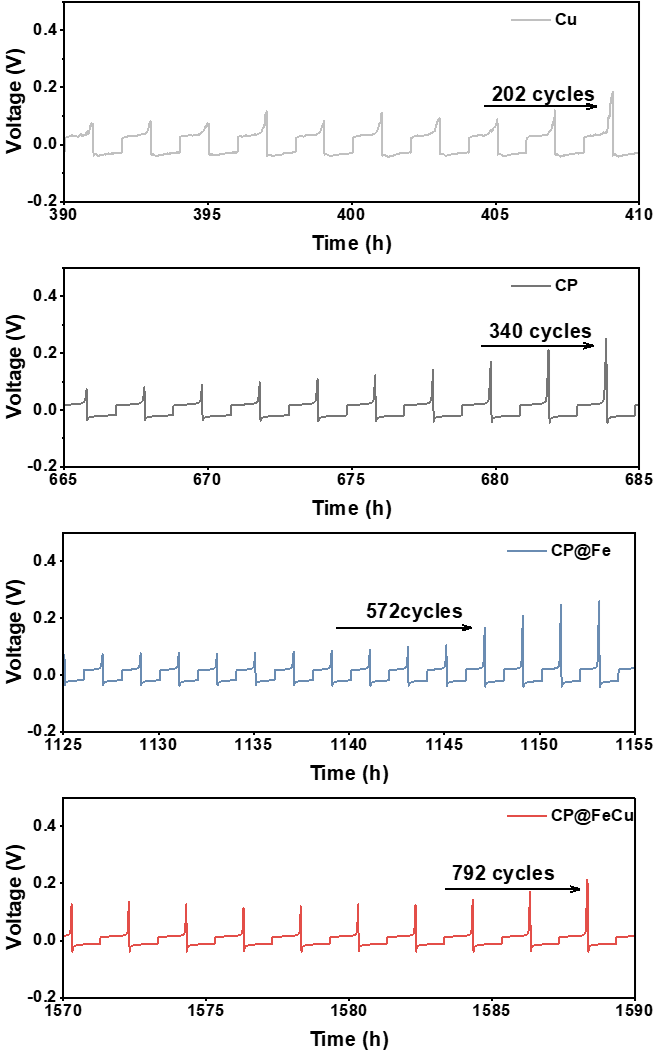


Figure S23. Galvanostatic profiles of the final cycles for special cycling stabilities of the Na metal anodes.


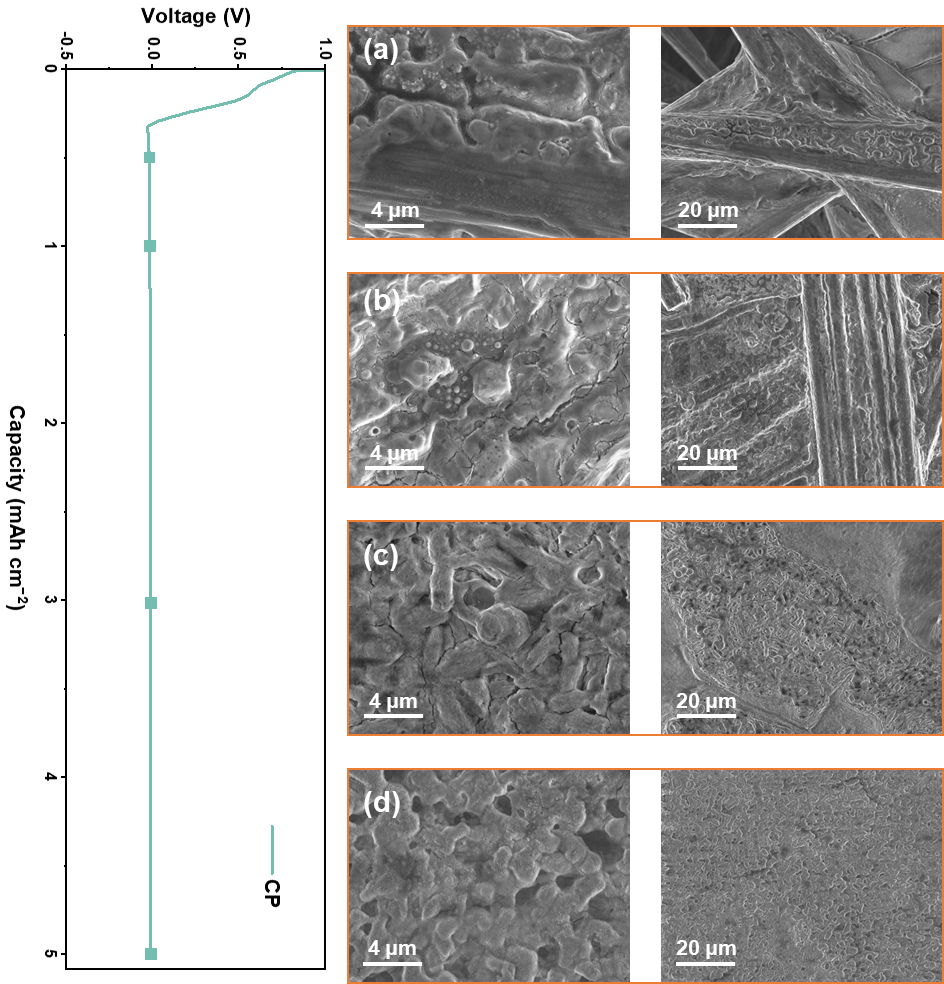


Figure S24. Voltage profile of Na deposition on CP electrodes at a current density of 1mA cm^−2^, and SEM images of the surface morphology of Na deposited at capacities of (a) 0.5 mA h cm^−2^, (b) 1 mA h cm^−2^, (c) 3 mA h cm^−2^ and (d) 5 mA h cm^−2^.


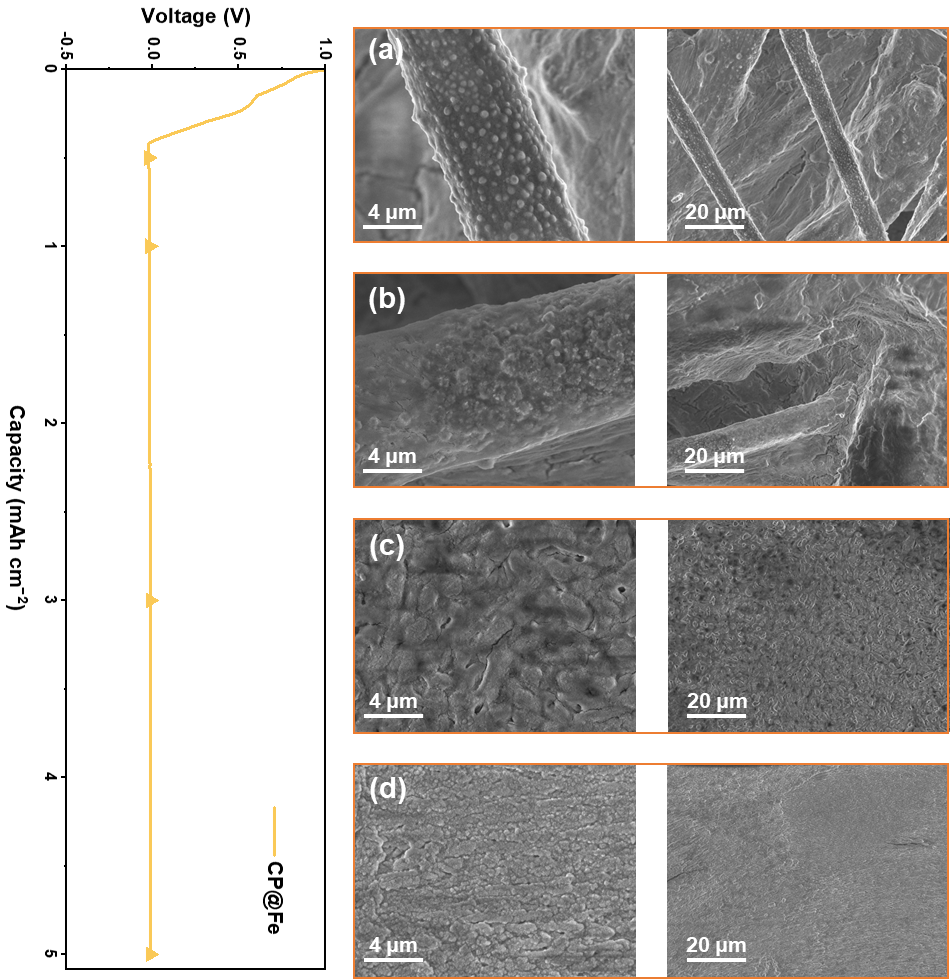


Figure S25. Voltage profile of Na deposition on CP@Fe electrodes at a current density of 1mA cm^−2^, and SEM images of the surface morphology of Na deposited at capacities of (a) 0.5 mA h cm^−2^, (b) 1 mA h cm^−2^, (c) 3 mA h cm^−2^ and (d) 5 mA h cm^−2^.


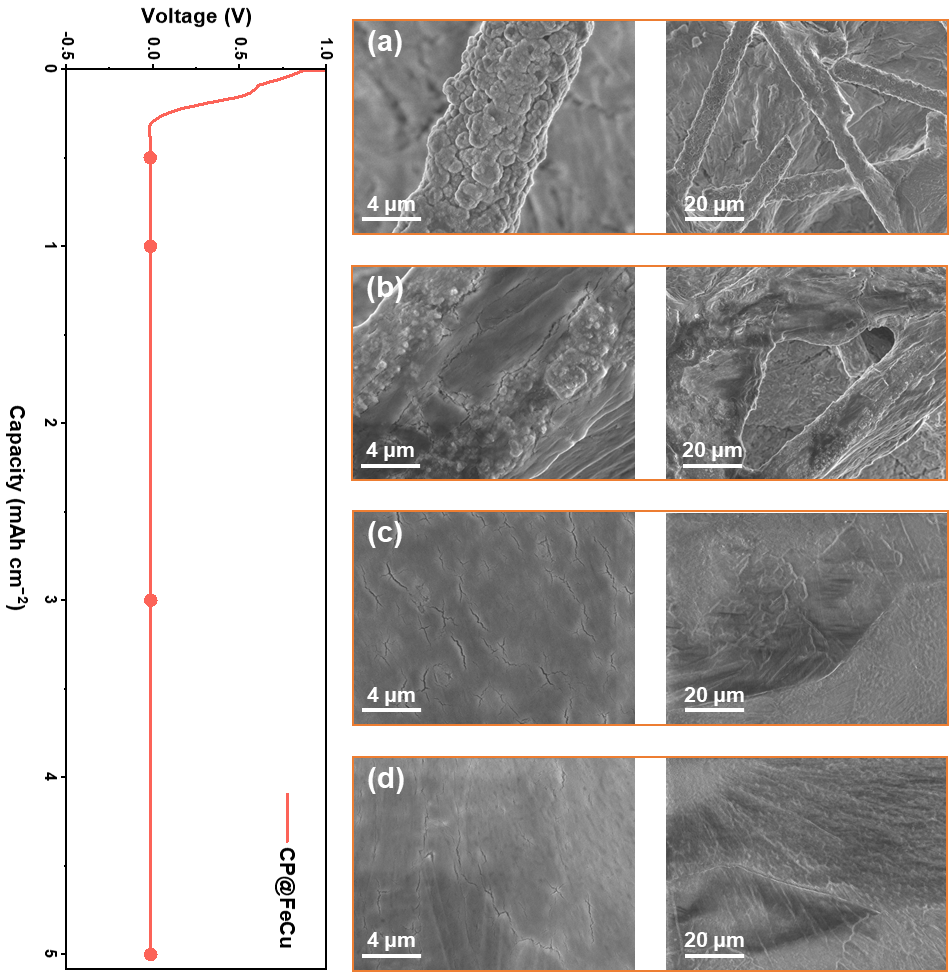


Figure S26. Voltage profile of Na deposition on CP@FeCu electrodes at a current density of 1mA cm^−2^, and SEM images of the surface morphology of Na deposited at capacities of (a) 0.5 mA h cm^−2^, (b) 1 mA h cm^−2^, (c) 3 mA h cm^−2^ and (d) 5 mA h cm^−2^.


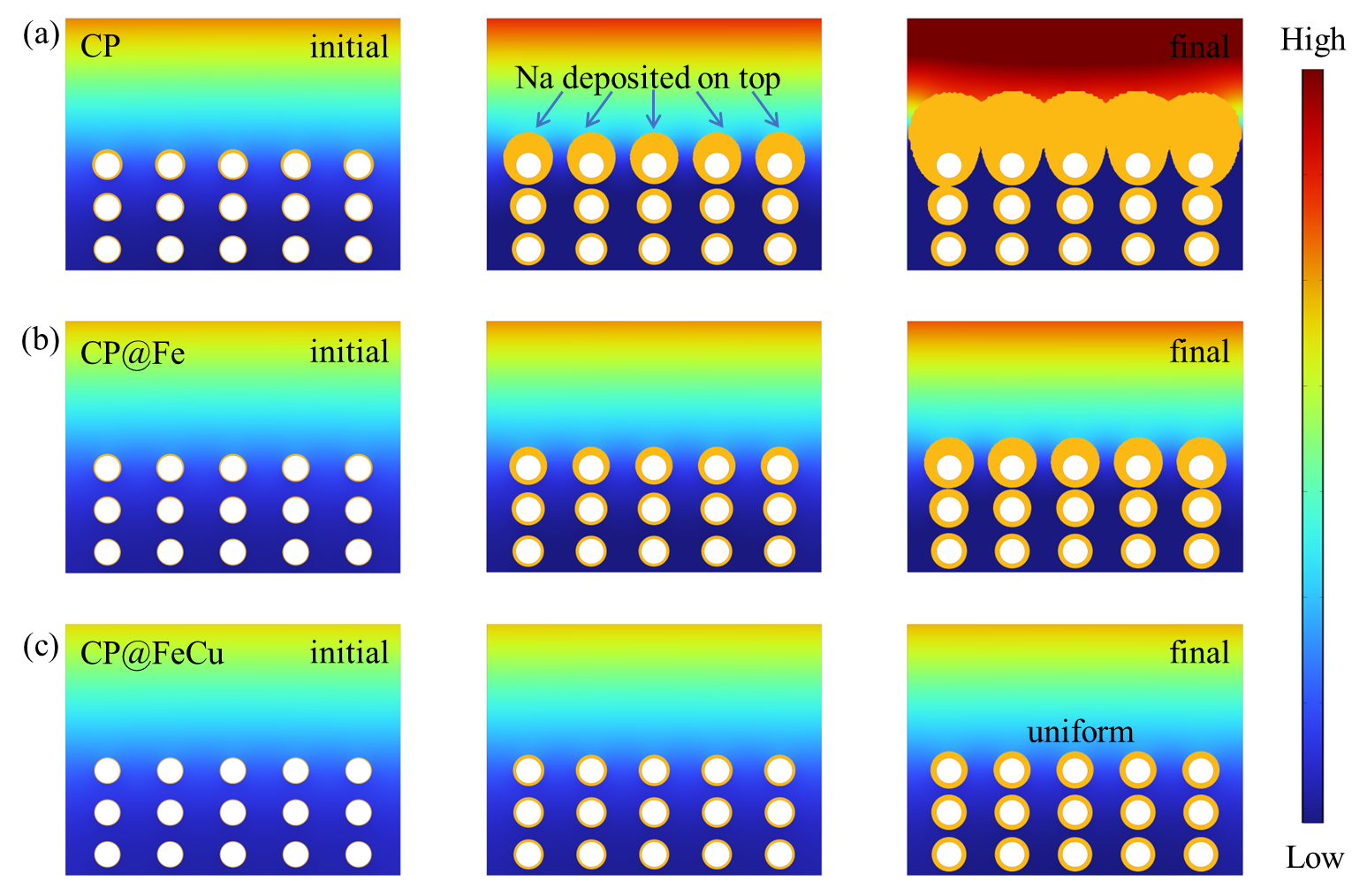


Figure S27. COMSOL Multiphysics simulations of the concentration distribution of sodium ions in (a) the CP, (b) CP@Fe, and (c) CP@FeCu hosts at various plating stages.

Figure S28. The deep discharge-charge curves of CP@Fe and CP@FeCu cathode-based Na-CO_2_ batteries at 5 μA cm^−2^ within a voltage range of 1.8–4.2 V versus Na/Na^+^.

Figure S29. Long-term cycling performances at 10 μA cm^−2^ and selected discharge and charge profiles of CP@FeCu cathode-based Na-CO_2_ battery during cycling.

**

Figure S30. The CV performance of Na-CO_2_ battery with CP@FeCu cathode at 0.1 mV s^−1^.

**

Figure S31. (a) Cycling performances and (b) corresponding time-voltage curves of Na-CO_2_ battery with CP@FeCu at 50 μA cm^−2^ and 100 μA cm^−2^.


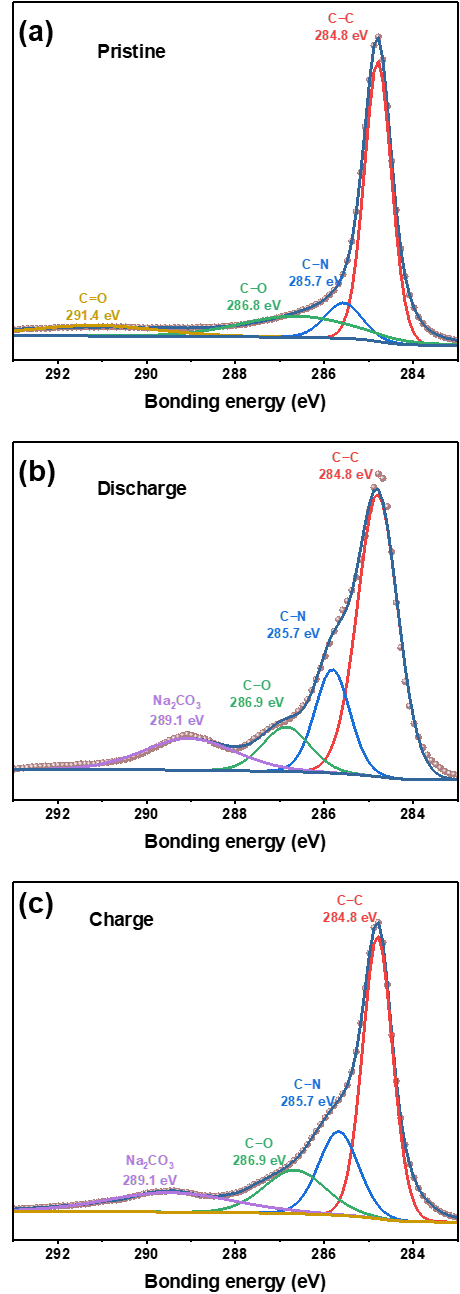


Figure S32. C1s XPS spectra of the CP@FeCu cathode at different stages: (a) pristine, (b) full discharge, and (c) charge process.


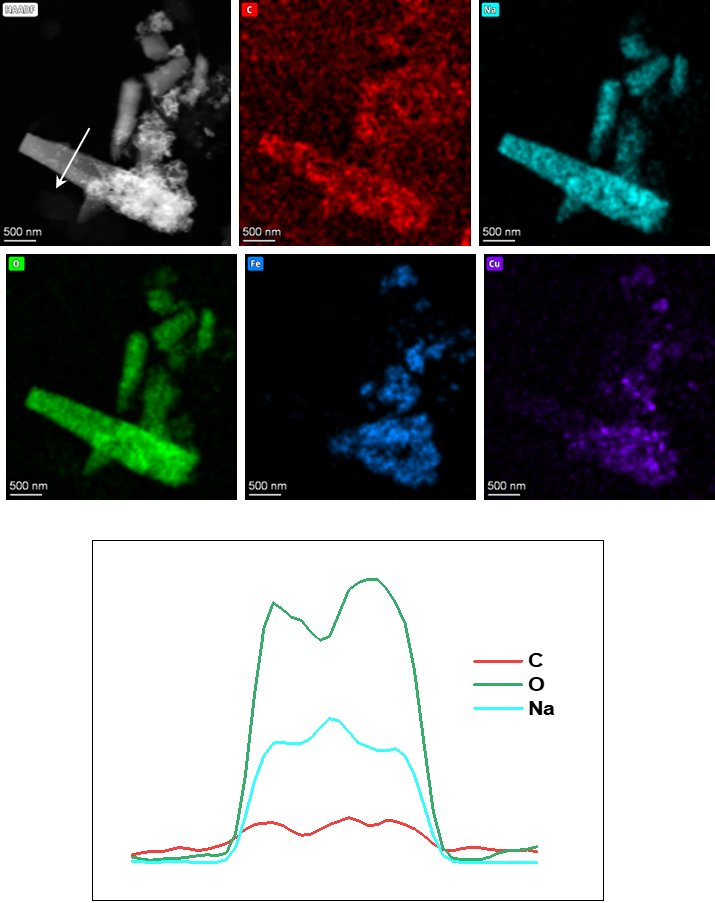


Figure S33. HAADF-STEM image and EDS maps, and corresponding line-scan profile of CP@FeCu cathode after discharge.

Figure S34. Fe 2p XPS spectra of CP@Fe and CP@FeCu.

Figure S35. XPS survey spectra of CP@FeCu.


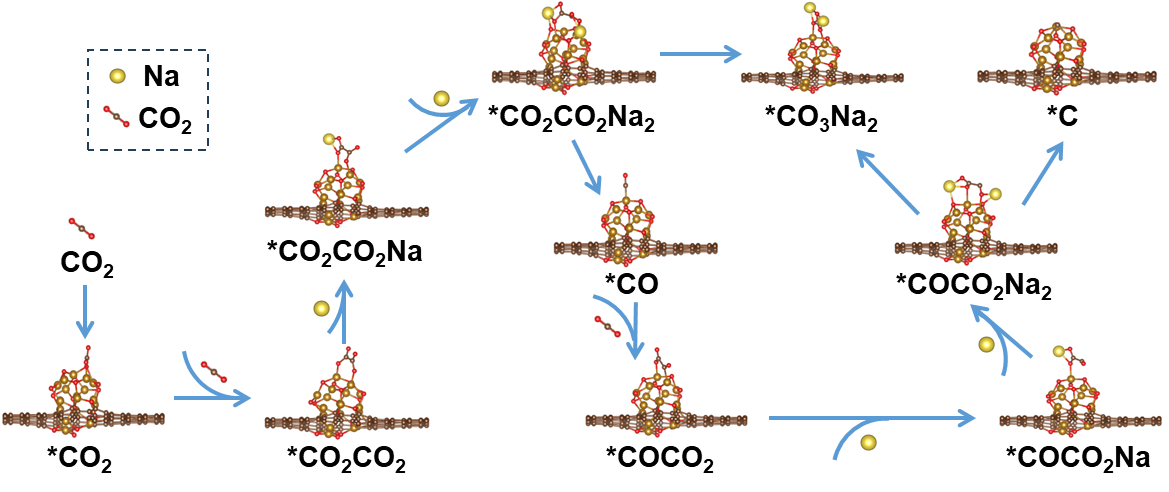


Figure S36.The reaction pathways and the optimized structures of intermediates and transition states on the CP@Fe interface.


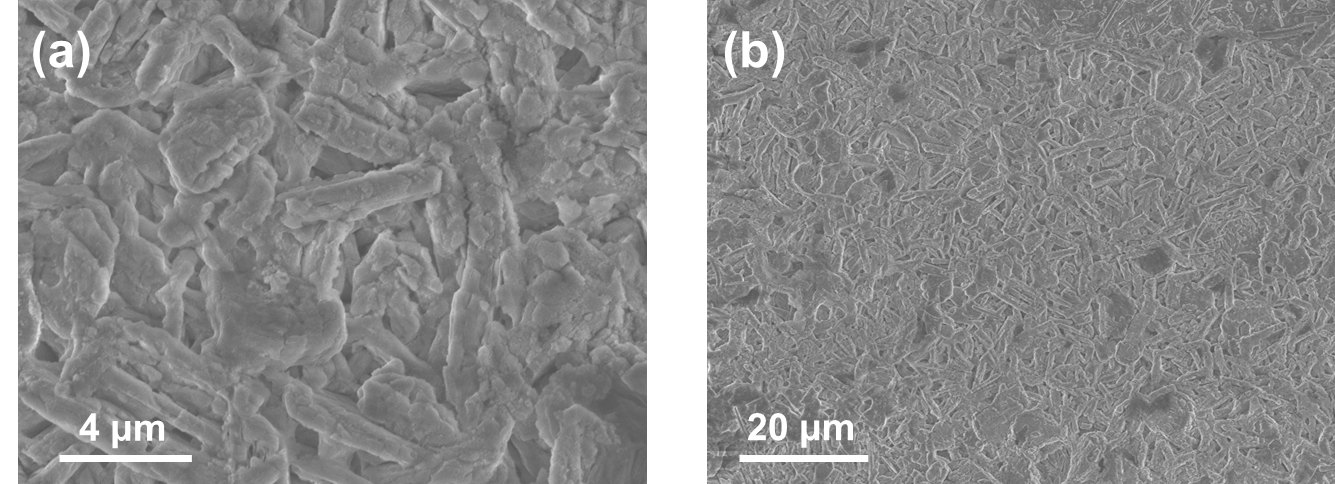


Figure S37. SEM images of the surface morphology of Na deposited on Cu foil at a capacity of 50 mA h cm^−2^.

**

Figure S38. The CV performance of (a) CP@FeCu-Na||CP@FeCu and (b) CP@Fe-Na||CP@Fe cells at 0.1 mV s^−1^.


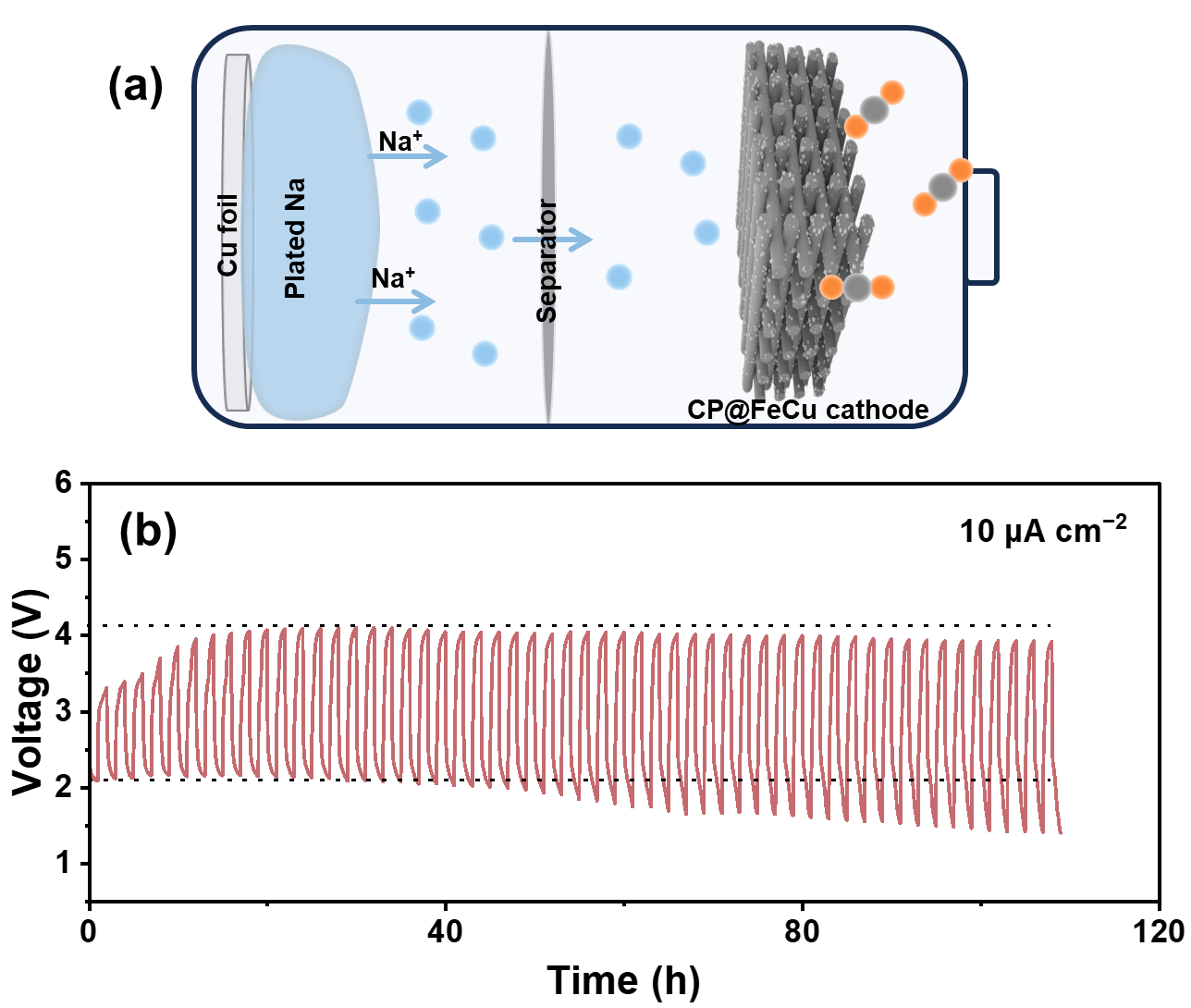


Figure S39. (a) Schematic of the cell configurations for anode-less Na-CO_2_ batteries employing CP@FeCu as CO_2_ cathode. (b) Long-term cycling performance at 10 μA cm^−2^.

Table S1 Electrochemical performance comparison of some reported sodiophilic materials with our work.

| **Materials** | **Electrolyte** | **Current density**  **(capacity)** | **Cumulative capacity**  **(cycle time)** | **Ref.** |
| --- | --- | --- | --- | --- |
| CP@FeCu | 1 M NaPF_6_ in diglyme | 1.0 mA cm^−2^  (1 mAh cm^−2^) | 1980 mAh cm^−2^  (3960 h) | **This work** |
| Fluorine-Super doped CNTs/Cellulose Nanofibrils Composite Paper (C_F_N-CP) | 1 M NaCF_3_SO_3_ in diglyme | 1.0 mA cm^−2^  (1 mAh cm^−2^) | 410 mAh cm^−2^  (820 h) | [7] |
| 3D carbon  microspheres interweaved fibers skeleton (CMFS) | 0.5 M NaCF_3_SO_3_ and 0.5 M NaPF_6_ in diglyme | 1.0 mA cm^−2^  (1 mAh cm^−2^) | 1400 mAh cm^−2^  (2800 h) | [8] |
| Toray TGP-H-060 | 1 M NaCF_3_SO_3_ in diglyme | 1.0 mA cm^−2^  (1 mAh cm^−2^) | 100 mAh cm^−2^  (200 h) | [9] |
| Uniform SnSe Nanoparticles on 3D Graphene Host (SnSe@GF) | 1 M NaCF_3_SO_3_ in diglyme | 1.0 mA cm^−2^  (1 mAh cm^−2^) | 1500 mAh cm^−2^  (3000 h) | [10] |
| Defect-rich carbon skeletons (L700) | 1 M NaCF_3_SO_3_ in diglyme | 1.0 mA cm^−2^  (2 mAh cm^−2^) | 600 mAh cm^−2^  (1200 h) | [11] |
| 3D hollow porous carbon nanofiber framework embedded with Sb nanoparticles (Sb@HPCNF) | 1 M NaPF_6_in diglyme | 1.0 mA cm^−2^  (1 mAh cm^−2^) | 200 mAh cm^−2^  (400 h) | [12] |
| Carbon nanofiber with Sn_4_P_3_ nanoparticles (Sn_4_P_3_NPs@CNF) | 1 M NaPF_6_in diglyme | 1.0 mA cm^−2^  (1 mAh cm^−2^) | 500 mAh cm^−2^  (1000 h) | [13] |
| Halloysite nanotubes with Ag nanoparticles (HNTs/Ag) | 1.0 M NaCF_3_SO_3_in DEGDME | 1.0 mA cm^−2^  (1 mAh cm^−2^) | 200 mAh cm^−2^  (400 h) | [14] |
| MXene@g-C_3_N_4_electrode | 1 M NaPF_6_in diglyme | 1.0 mA cm^−2^  (1 mAh cm^−2^) | 300 mAh cm^−2^  (600 h) | [15] |
| SnO_2_/Ti_3_C_2_T_x_ | 1 M NaPF_6_in diglyme | 1.0 mA cm^−2^  (1 mAh cm^−2^) | 700 mAh cm^−2^  (1400 h) | [16] |
| A porous nitrogen-anchored carbon embedded with Co nanoparticles (CoNC) | 1 M NaCF_3_SO_3_ in DME | 1.0 mA cm^−2^  (1 mAh cm^−2^) | 400 mAh cm^−2^  (800 h) | [17] |
| Sn@LIG@Cu | 1 M NaPF_6_in diglyme | 1.0 mA cm^−2^  (1 mAh cm^−2^) | 1300 mAh cm^−2^  (2600 h) | [18] |
| PVDF@Cu current collector | 1 M NaPF_6_ in diglyme | 1.0 mA cm^−2^  (1 mAh cm^−2^) | 1000 mAh cm^−2^  (2000 h) | [19] |
| Molecular-designed porous triazine framework with integrated F functionality  (FCTF) coated Cu | 1 M NaPF_6_in diglyme | 1.0 mA cm^−2^  (1 mAh cm^−2^) | 1200 mAh cm^−2^  (2400 h) | [20] |
| Cu foam skeleton with  hierarchical ZnO nanorod arrays (CF@ZnO) | 1.0 M NaCF_3_SO_3_in DEGDME | 1.0 mA cm^−2^  (1 mAh cm^−2^) | 300 mAh cm^−2^  (600 h) | [21] |
| Carbon-encapsulated mosaic Fe_3_O_4_ nanosheets (Fe_3_O_4_@CNS) | 1.5 M NaCF_3_SO_3_ in diglyme | 1.0 mA cm^−2^  (1 mAh cm^−2^) | 900 mAh cm^−2^  (1800 h) | [22] |
| Fe_7_S_8_@CNS | 1.5 M NaCF_3_SO_3_ in diglyme | 1.0 mA cm^−2^  (1 mAh cm^−2^) | 1000 mAh cm^−2^  (2000 h) | [23] |

Table S2 Comparison of Aprotic Na-CO_2_ batteries

| **Working conditions** | **Electrolyte** | **Cathode materials** | **Voltage gap,**  **applied current** | **Cyclability** | **Ref.** |
| --- | --- | --- | --- | --- | --- |
| Aprotic | 1.0 M NaClO_4_/TEGDME | CP@FeCu | ∼1.65 V, 5 μA cm^−2^ | 2400 cycles | **This work** |
| Aprotic | 0.5 M NaPF_6_/TEGDME | CNTs + LCO | ∼3.65 V, 400 mA g^−1^ | 50 cycles | [24] |
| Aprotic | 1.0 M NaClO_4_/TEGDME | γ-MnO_2_ | 1.18V, 50 mA g^−1^ | 50 cycles | [25] |
| All-solid-state | NASICON electrolyte | Succinonitrile-treated Ru/CNTs | ~1.3 V, 50 mA g^−1^ | 70 cycles | [26] |
| Aprotic | 1 M NaClO_4_/ TEGDME | CMO@CF | ~2.0 V, 200 mA g^−1^ | 75 cycles | [27] |
| All-solid-state | Polymer electrolyte of PEO-NaClO_4_/glass fiber matrix | NC900 | ~1.5 V, 100 mA g^−1^ | 80 cycles | [28] |
| Aprotic | NaF, NaNO_3_, NaOTF/ Diglyme | N-doped nanocarbon | ~1.5V, 200 mA g^−1^ | 100 cycles | [29] |
| All-solid-state | SN-based electrolyte (compact NaF-rich interphase on Na surface) | MWCNTs | ~1.53 V, 50 mA g^−1^ | 100 cycles | [30] |
| Aprotic | 1 M NaClO_4_/ TEGDME | MoS_2_/SnS_2_ | ~1 V, 50 mA g^−1^ | 100 cycles | [31] |
| All-solid-state | PVDF−HFP/ Na_3.2_Zr_1.9_Mg_0.1_Si_2_PO_12_ | Ru-CNTs | ~2 V, 200 mA g^−1^ | 120 cycles | [32] |
| Aprotic | 1 M NaClO_4_/ TEGDME | RuO_2_@a-MWCNTs | ~1.2V, 100 mA g^−1^ | 120 cycles | [33] |
| Aprotic | 1 M NaClO_4_/ TEGDME | Ru@KB | ~1.5 V, 100 mA g^−1^ | 130 cycles | [34] |
| Aprotic | 1 M NaClO_4_/ TEGDME | ZnCo_2_O_4_@CNT | ~1.8 V, 100 mA g^−1^ | 150 cycles | [35] |
| Aprotic | 1 M NaClO_4_/ TEGDME | TEGDME-treated MWCNT (t-MWCNT) | ~1.00 V, 1000 mA g^−1^ | 200 cycles | [36] |
| Flexible solid-state | SPE consisting of PEO/NaClO_4_/3 wt% SiO_2_ | MWCNTs@Ni | ~1.2 V, 50 mA g^−1^ | 240 cycles | [37] |
| Quasi-solid-state | NASICON, gel electrolyte | Co-NCF | ~1.75 V, 0.1 mA cm^−2^ | 367 cycles | [38] |
| Aprotic, (sodium-fluorinated graphene anode) | 0.5 M NaCF_3_SO_3_/ TEGDME | carbon cloth-supported δ-MnO_2_ electrodes | ~1.5 V, 200 mA g^−1^ | 391 cycles | [39] |
| Quasi-solid (Reduced graphene oxide Na anodes) | CPE consisting of PVDF-HFP-4% SiO_2_/NaClO_4_–TEGDME | TEGDME activated MWCNT  (a-MCNTs) | ~1.75 V, 500 mA g^−1^ | 400 cycles | [40] |

References

[1] M. Linck, P. Hartel, S. Uhlemann, F. Kahl, H. Müller, J. Zach, M. Haider, M. Niestadt, M. Bischoff, J. Biskupek, Z. Lee, T. Lehnert, F. Börrnert, H. Rose, U. Kaiser, *Phys. Rev. Lett.* **2016**, *117*, 076101.

[2] B. D. Adams, J. Zheng, X. Ren, W. Xu, J. G. Zhang, *Adv. Energy Mater.* **2017**, *8*, 1702097.

[3] G. Kresse, J. Furthmuller, *Phys. Rev. B* **1996**, *54*, 11169.

[4] G. Kresse, J. Furthmuller, *Comp. Mater. Sci.* **1996**, *6*, 15.

[5] J. A. White, D. M. Bird, *Phys. Rev. B* **1994**, *50*, 4954.

[6] J. P. Perdew, K. Burke, M. Ernzerhof, *Phys. Rev. Lett.* **1996**, *77*, 3865.

[7] J. Xiao, N. Xiao, K. Li, L. Zhang, X. Ma, Y. Li, C. Leng, J. Qiu, *Adv. Funct. Mater.* **2022**, *32*, 2111133.

[8] L. Yue, Y. Qi, Y. Niu, S. Bao, M. Xu, *Adv. Energy Mater.* **2021**, *11*, 2102497.

[9] Q. Zhang, Y. Lu, M. Zhou, J. Liang, Z. Tao, J. Chen, *Inorg. Chem. Front.* **2018**, *5*, 864.

[10] M. Y. Xu, Z. J. Liu, Y. Li, N. Mubarak, H. L. Wong, M. Tamtaji, Y. H. Zhao, Y. Y. Li, J. Wang, J. W. You, H. W. Liu, Y. T. Cai, K. A. Zhang, F. Xu, K. Amine, J. K. Kim, Z. T. Luo, *Energy Storage Mater.* **2023**, *60*, 102848.

[11] Z. Xu, Z. Guo, R. Madhu, F. Xie, R. Chen, J. Wang, M. Tebyetekerwa, Y.-S. Hu, M.-M. Titirici, *Energy Environ. Sci.* **2021**, *14*, 6381.

[12] Z. Li, H. Qin, W. Tian, L. Miao, K. Cao, Y. Si, H. Li, Q. Wang, L. Jiao, *Adv. Funct. Mater.* **2023**, *34*, 2301554.

[13] Y. J. Liu, M. Bai, D. Du, X. Y. Tang, H. L. Wang, M. Zhang, T. Zhao, F. Liu, Z. Q. Wang, Y. Ma, *Energy Environ. Mater.* **2023**, *6*, e12350.

[14] C. Yang, Y. Zhang, Y. Hua, H. Wang, A. Tang, H. Yang, *ACS Appl. Mater. Interfaces* **2023**, *15*, 11949.

[15] C. Y. Bao, J. H. Wang, B. Wang, J. G. Sun, L. C. He, Z. H. Pan, Y. P. Jiang, D. L. Wang, X. M. Liu, S. X. Dou, J. H. Wang, *ACS Nano* **2022**, *16*, 17197.

[16] Z. P. Li, Y. M. Zhang, H. T. Guan, S. K. Meng, Y. F. Lu, J. Wang, G. S. Huang, X. Li, J. Q. Cui, Q. Li, Q. C. Zhang, B. H. Qu, *Small* **2023**, *19*, 2208277.

[17] Y. Y. Xie, J. X. Hu, Z. X. Han, T. S. Wang, J. Q. Zheng, L. Gan, Y. Q. Lai, Z. A. Zhang, *Energy Storage Mater.* **2020**, *30*, 1.

[18] H. Xiao, Y. Li, W. Chen, T. Xie, H. Zhu, W. Zheng, J. He, S. Huang, *Small* **2023**, e2303959.

[19] Z. Hou, W. H. Wang, Y. K. Yu, X. X. Zhao, Q. W. Chen, L. F. Zhao, Q. Di, H. X. Ju, Z. W. Quan, *Energy Storage Mater.* **2020**, *24*, 588.

[20] R. Zhuang, X. Zhang, C. Qu, X. Xu, J. Yang, Q. Ye, Z. Liu, S. Kaskel, F. Xu, H. Wang, *Sci. Adv.* **2023**, *9*, eadh8060.

[21] W. Yang, W. Yang, L. Dong, G. Shao, G. Wang, X. Peng, *Nano Energy* **2021**, *80*, 105563.

[22] P. Liu, X. Wang, X. Jia, J. Zhou, *ACS Appl. Mater. Interfaces* **2022**, *14*, 35873.

[23] R. L. Yuan, P. Liu, X. M. Wang, J. S. Zhou, *Small* **2023**, *19*, 2300919.

[24] W. Huang, J. Qiu, Y. Ji, W. Zhao, Z. Dong, K. Yang, M. Yang, Q. Chen, M. Zhang, C. Lin, K. Xu, L. Yang, F. Pan, *ACS Nano* **2023**, *17*, 5570.

[25] X. Chen, J. Chen, Y. Liu, Y. Liu, Y. Gao, S. Fan, X. He, X. Liu, C. Shen, Y. Jiang, L. Li, Y. Qiao, S. Chou, *ACS Appl. Mater. Interfaces* **2023**, *15*, 28106.

[26] Z. Tong, S. B. Wang, M. H. Fang, Y. T. Lin, K. T. Tsai, S. Y. Tsai, L. C. Yin, S. F. Hu, R. S. Liu, *Nano Energy* **2021**, *85*, 105972.

[27] C. Fang, J. Luo, C. Jin, H. Yuan, O. Sheng, H. Huang, Y. Gan, Y. Xia, C. Liang, J. Zhang, W. Zhang, X. Tao, *ACS Appl Mater Interfaces* **2018**, *10*, 17240.

[28] X. Hu, P. H. Joo, E. Matios, C. Wang, J.-m. Luo, K. Yang, W. Li, *Nano Lett.* **2020**, *20*, 3620.

[29] X. Hu, Y. Zhang, P. Wang, E. Matios, W. Li, *ACS Nano* **2022**, *16*, 17965.

[30] Y. Lu, Y. Cai, Q. Zhang, L. Liu, Z. Niu, J. Chen, *Chem. Sci.* **2019**, *10*, 4306.

[31] K. Pichaimuthu, A. Jena, H. Chang, C. Su, S. Hu, R. Liu, *ACS Appl. Mater. Interfaces* **2022**, *14*, 5834.

[32] L. Lu, C. Sun, J. Hao, Z. Wang, S. F. Mayer, M. T. Fernández‐Díaz, J. A. Alonso, B. Zou, *Energy Environ. Mater.* **2022**, *6*, e12364.

[33] Z. Wang, Y. Cai, Y. Ni, Y. Lu, L. Lin, H. Sun, H. Li, Z. Yan, Q. Zhao, J. Chen, *Chin. Chem. Lett.* **2022**, *34*, 107405.

[34] L. Guo, B. Li, V. Thirumal, J. Song, *Chem. Commun.* **2019**, *55*, 7946.

[35] S. Thoka, Z. Tong, A. Jena, T. Hung, C. Wu, W. Chang, F. Wang, X. Wang, L. Yin, H. Chang, S. Hu, R. Liu, *J. Mater. Chem. A* **2020**, *8*, 23974.

[36] X. Hu, J. Sun, Z. Li, Q. Zhao, C. Chen, J. Chen, *Angew. Chem. Int. Ed.* **2016**, *55*, 6482.

[37] X. Wang, X. Zhang, Y. Lu, Z. Yan, Z. Tao, D. Jia, J. Chen, *ChemElectroChem* **2018**, *5*, 3628.

[38] B. W. Xu, D. Zhang, S. L. Chang, M. J. Hou, C. Peng, D. F. Xue, B. Yang, Y. Lei, F. Liang, *Cell Rep Phys Sci* **2022**, *3*, 100973.

[39] Y. Mao, X. Chen, H. Cheng, Y. Lu, J. Xie, T. Zhang, J. Tu, X. Xu, T. Zhu, X. Zhao, *Energy Environ. Mater.* **2021**, *5*, 572.

[40] X. Hu, Z. Li, Y. Zhao, J. Sun, Q. Zhao, J. Wang, Z. Tao, J. Chen, *Sci. Adv.* **2017**, *3*, 1602396.
